# Metabolic profiling and transcriptome analysis provide insights into the accumulation of flavonoids in chayote fruit during storage

**DOI:** 10.3389/fnut.2023.1029745

**Published:** 2023-02-27

**Authors:** YuTing Pu, Cheng Wang, YongWen Jiang, XiaoJing Wang, YuJie Ai, WeiBing Zhuang

**Affiliations:** ^1^Key Laboratory of Plant Resource Conservation and Germplasm Innovation in Mountainous Region, Ministry of Education, Collaborative Innovation Center for Mountain Ecology and Agro-Bioengineering (CICMEAB), College of Life Sciences, Institute of Agro-Bioengineering, Guizhou University, Guiyang, Guizhou, China; ^2^Hubei Key Laboratory of Quality Control of Characteristic Fruits and Vegetables, College of Life Science and Technology, Hubei Engineering University, Xiaogan, China; ^3^Tea Research Institute, Chinese Academy of Agricultural Sciences (CAAS), Hangzhou, China; ^4^The Jiangsu Provincial Platform for Conservation and Utilization of Agricultural Germplasm, Jiangsu Province and Chinese Academy of Sciences, Nanjing Botanical Garden Memorial Sun Yat-sen, Institute of Botany, Nanjing, China

**Keywords:** *Sechium edulel*, metabolite profiling, transcriptome profiling, flavonoids, DEGs

## Abstract

Chayote (*Sechium edulel*) fruits are rich in flavonoids, folate, and low-calorie food. However, studies about the flavonoids and the corresponding regulatory mechanism of flavonoid synthesis in chayote fruits was still unclear. In present study, an integrated transcriptome and metabolite analysis of chayote fruits at three different storage stages were conducted to explore the flavonoid compositions and gene expression associated with flavonoid synthesis. Through the UPLC-MS/MS analysis, a total of 57 flavonoid compounds were detected. Of these, 42 flavonoid glycosides were significantly differential accumulation in chayote fruits at three different storage stages. Many genes associated with flavonoid synthesis were differentially expressed in chayote fruits at three different storage stages through RNA-seq analysis, including structural genes and some TFs. There was a high correlation between RNA-seq analysis and metabolite profiling, and the expression level of candidate genes in the flavonoid synthesis pathway were consistent with the dynamic changes of flavonoids. In addition, one R2R3-MYB transcription factor, FSG0057100, was defined as the critical regulatory gene of flavonoid synthesis. Furthermore, exogenous application of phenylalanine increased the total content of flavonoids and promoted some flavonoid biosynthesis-related gene expression in chayote fruits. The above results not only make us better understand the molecular mechanism of flavonoid synthesis in chayote fruits, but also contribute to the promotion and application of chayote products.

## 1. Introduction

*Sechium edulel* (Jacq.) Swartz (chayote), an herbaceous perennial climbing plant, is cultivated in most tropical and subtropical areas (1–4). The chayote fruits are not only rich in nutrients, such as amino acids, vitamins, α-aminobutyric acid, and dietary fiber, but also contain a lot of flavonoids (5–9). Flavonoids are the low molecular weight polyphenolic secondary metabolic compounds produced by plants, which play important roles in human health benefits (10, 11). Thus, research on flavonoid synthesis in chayote fruits has won attracted considerable attention.

Flavonoids, synthesized by the phenylpropanoid pathway, played important roles in extending the shelf life of fruits and vegetables (12). Structure genes encoding key enzymes associated with the flavonoid synthesis, including phenylalanine ammonia lyase (PAL), 4-coumarate-CoA ligase (4CL), cinnamate 4-hydroxylase (C4H), chalcone isomerase (CHI), chalcone synthase (CHS), flavonoid 3′-hydroxylase (F3′H), isoflavone synthase (IFS), flavanone 3-hydroxylase (F3H), dihydroflavonol 4-reductase (DFR), flavonol synthase (FLS), anthocyanidinreductase (ANR), anthocyanidin synthase (ANS), and leucoanthocyanidin reductase (LAR). The functions of corresponding structure genes have been well characterized in various kinds of plants, such as *Arabidopsis* (13), maize (*Zea mays*) (14), rice (*Oryza sativa*) (15), Melon (*Cucumis melo*) (16), cucumber (*Cucumis sativus*) (17). Although previous studies reported that flavonoids were widely distributed in all parts of *S. edulel* plant, such as leaves, stems, roots and chayote fruits (7), the detailed composition of flavonoids and the corresponding molecular regulatory mechanism in chayote fruits during storage were still unclear.

Flavonoids have garnered considerable attention because of numerous nutritional and biological benefits, which were often applied in food processing to help prolong the shelf-life of food items due to their antioxidant property (18). Previous studies revealed that the total phenolic contents of rowanberry (*Sorbus aucuparia*) fruits and banana (*Musa nana*) significantly reduced during the 20-or 2-day storage at 22°C, respectively (19, 20). Aziz et al. (21) investigated the contents changes of flavonoids in *Cerasus Humilis* fruits during storage revealed that the total content of flavonoids was reduced during storage. Robles-Sánchez et al. (22) researched the contents changes of flavonoids in mangoes during Low-Temperature storage revealed that the total content of flavonoids was reduced during the 15-day storage. Chayote has a short shelf life, which can sprout in 4–6 weeks if the storage conditions allowed (23). However, the dynamic changes of flavonoids contents and corresponding molecular mechanism during shelf life was still unclear.

Combination transcriptomic and metabolomic analyses may be a good approach to to explore the functions of genes associated with metabolism (24). Maoz’s teams explored the response of table grapes under storage at anaerobic and cold stresses with an integrated transcriptomic and metabolomic analysis (25), and found that genes associated with phenylpropanoid pathway and pyruvate metabolism were differentially expressed under storage at anaerobic and cold stresses. A transcriptome and metabolome profiling were performed to explore the bluing response of radish roots under storage, and found that low reduction system: ascorbic acid (ASA), glutathione (GSH), ascorbate peroxidase (APX), glutathione peroxidase (GPX), glutathione-S-transferase (GST), or high content of glucosinolates, oxidation system: reactive oxygen species (ROS), catalase (CAT), peroxidase (POD) could promote the blue discoloration of radish roots (26). LTC condition is the prestorage at 8°C for 5 days before storage at 0°C, and Wang’s teams conducted the integrated analysis of transcript and metabolite profiling of ‘Hujingmilu’ peach stored at 0°C conditions and LTC conditions. The results indicated that LTC can relieve chilling injury in peach fruit (27). Although the chayote fruit quality deteriorates with storage, the dynamic changes of the flavonoids in chayote fruits at room temperature for a fairly long time was still unclear. Thus, in present study, the chayote fruits placed in plastic containers at room temperature was used to perform the transcriptome and metabolome analysis.

In this study, we here performed metabolome and transcriptome profiling of chayote fruits during storage at room temperature to examine the dynamic changes of flavonoid compounds and the corresponding regulatory mechanism of flavonoid synthesis. A total of 57 flavonoid derivatives were detected in this study. Among them, 42 flavonoid glycosides were differentially accumulated in different storage stages. Moreover, the DEGs, including structural genes and transcriptional factors (TFs) for flavonoid synthesis were also identified, and the expression pattern of some DEGs were further verified using qRT-PCR. Correlation analysis between RNA-seq analysis and metabolite profiling had revealed that the identified structural genes and TFs displayed better correlation with the quantitative changes of flavonoids. This study not only makes us better understand the molecular mechanism of flavonoid synthesis in chayote fruits, but also contributes to the promotion and application of chayote products.

## 2. Materials and methods

### 2.1. Plant material, growth conditions, and treatments

The Chayote was purchased from a local supplier (Walmart, Huaxi District, Guiyang City, Guizhou Province, China) at their commercial maturity stage, and then planted in the experimental field of Guizhou university. The mature chayote fruits with the same size were collected from the experimental field of Guizhou University in late October 2021. To avoid excessive dehydration, all chayote fruit samples were collected and placed in plastic containers at optimum storage temperature (10°C) for 1 month (28). Three storage stages (S1, optimal harvest stage; S2, 15 days after storage; S3, 30 days after storage) were collected to perform RNA-seq analysis and metabolome profiling, respectively. Three independent biological replicates were performed.

### 2.2. Phenylalanine treatment

The mature chayote fruits with the same age and size were collected on October 10, 2021 and used for phenylalanine treatment. The phenylalanine concentration was determined based on Watanabe’s research (29), and the operation steps are as follows: the cottons containing phenylalanine solution with different concentrations (0.5-and 1 mM) were wrapped on the surface of fruits. The cotton was changed every 3 days. The cotton-wrapped chayote fruits were is placed in plastic containers at optimum storage temperature (10°C) for 10 days. After 10 days of treatments, the chayote fruits were sampled and immediately placed into liquid nitroge.

### 2.3. Total flavonoid measurement and metabolite extraction

Determination of total flavonoid contents in chayote at three different stages (S1 ~ S3) was conducted using the Plant Flavonoids Content Assay Kit (Beijing Boxbio Science & Technology Co., Ltd.). The frozen chayote were crushed to fine powder using grinding bead (6 mm diameter, 6 min, 50 Hz, MM400, Retsch). Fifty milligrams of fine-powdered samples were extracted from the ultrasonic and cryogenic instrumentation at 5°C (40 KHz) using 400 μL of 70% aqueous methanol. The supernatants were collected by centrifugation (13,000 g, 15 min) and then passed through a hydrophilic nylon membrane filter (Pore size: 0.22 μM). Pipette 20 μL of supernatant for each sample and mix them as the quality control samples (QC).

### 2.4. Untargeted metabolite profiling analysis

Metabolite profiling analysis of the extracted samples was conducted using UPLC-MS/MS method (30). Chromatographic separation of flavonoid metabolites was conducted on an ACQUITY UPLC HSS T3 column (100 mm × 2.1 mm, 1.8 μM) using mobile phases A (5% acetonitrile in water contained 0.1% formic acid) and mobile phases B (47.5% acetonitrile in 47% isopropanol solution contained 0.1% formic acid). The elution profile was set as follows: 0–2.5 min, 95:5 phase A/phase B to 75:25 phase A/phase B; 2.5–9 min, 75:25 phase A/phase B to 5:95 phase A/phase B; 9–13 min, 5:95 phase A/phase B to 5:95 phase A/phase B; 13–13.1 min, 5:95 phase A/phase B to 95:5 phase A/phase B, 13.1–16 min, 95:5 phase A/phase B to 95:5 phase A/phase B. The flow rate was 0.4 ml/min. The column temperature was maintained at 40°C. The injection volume was 10 μL.

The quadrupole-time-of-flight mass spectrometer (MS) was used to profile the metabolites of fruit samples (31). The analytical MS parameters were as follows: source temperature, 550°C; ion sources GS1, GS2, and curtain gas were set as 50, 50 and 30 psi, respectively; collision energy (CE), declustering potential (DP), ion-spray voltage floating in negative mode, and in positive mode were set as 20 ~ 60 V, 80 V, 4 kV, and 5 kV. MS acquisition was performed in a data-dependent acquisition (DDA) mode.

### 2.5. Qualitative and quantificatiive analysis of metabolites

Data preprocessing were performed by Progenesis QI software to obtain the m/z values, retention time (RT), and peak intensity. Metabolites were found by searching and comparing the m/z values, RT, and fragmentation patterns to standards in Human metabolome database (HMDB)^1^ and Metlin database.^2^ The variable importance of the projection (VIP) score of OPLS-DA model was performed to detect global metabolic changes among comparable groups. Metabolites with *t* test *p*-value <0.05 and VIP ≥ 1 were defined as differential metabolites (32). The R package from Bioconductor on Majorbio Cloud Platform^3^ was used to perform multivariate statistical analysis. An unsupervised method was used to perform principal component analysis (PCA) (33).

### 2.6. RNA-seq analysis

Extraction of the total RNA from chayote fruits at three different storage stages (S1 ~ S3) was performed using the EAZY spin Plus Plant RNA Kit. The mRNA purification, library construction, and RNA-seq was conducted by Majorbio Technology Co., Ltd (Shanghai, China). The heatmap was drawn with the log2-RPKM of individual DEGs. The RNA-seq data set is available in the Gene Expression Omnibus (accession no. GSE214641).

### 2.7. Quantitative real-time polymerase chain reaction

The total RNA was extracted from chayote fruits at S1, S2, and S3 stages and phenylalanine-treated seedlings using with TaKaRa MiniBEST Plant RNA Extraction Kit (TaKaRa) and the corresponding cDNA was obtained with cDNA Reverse Transcription Kit (TaKaRa). qRT-PCR was conducted with SYBR Premix Ex Tag on a DNA Engine Opticon™ 2 system (Bio-Rad Laboratories Inc.). AUG gene was used to normalize gene expression of candidate genes. A list of primers used in this study were presented in Table S1. The expression levels were evaluated by the 2^−ΔΔCt^ method with three biological replicates.

### 2.8. Statistical analysis

Significant analysis between two sample groups were calculated using T-test analysis (34). All of expression analysis were conducted for three biological replicates. The average data of three biological replicates was displayed with plus or minus the standard deviation (average ± SD).

## 3. Results

### 3.1. Measurement of total flavonoid content in chayote fruits at three different storage stages

**Figure 1:**
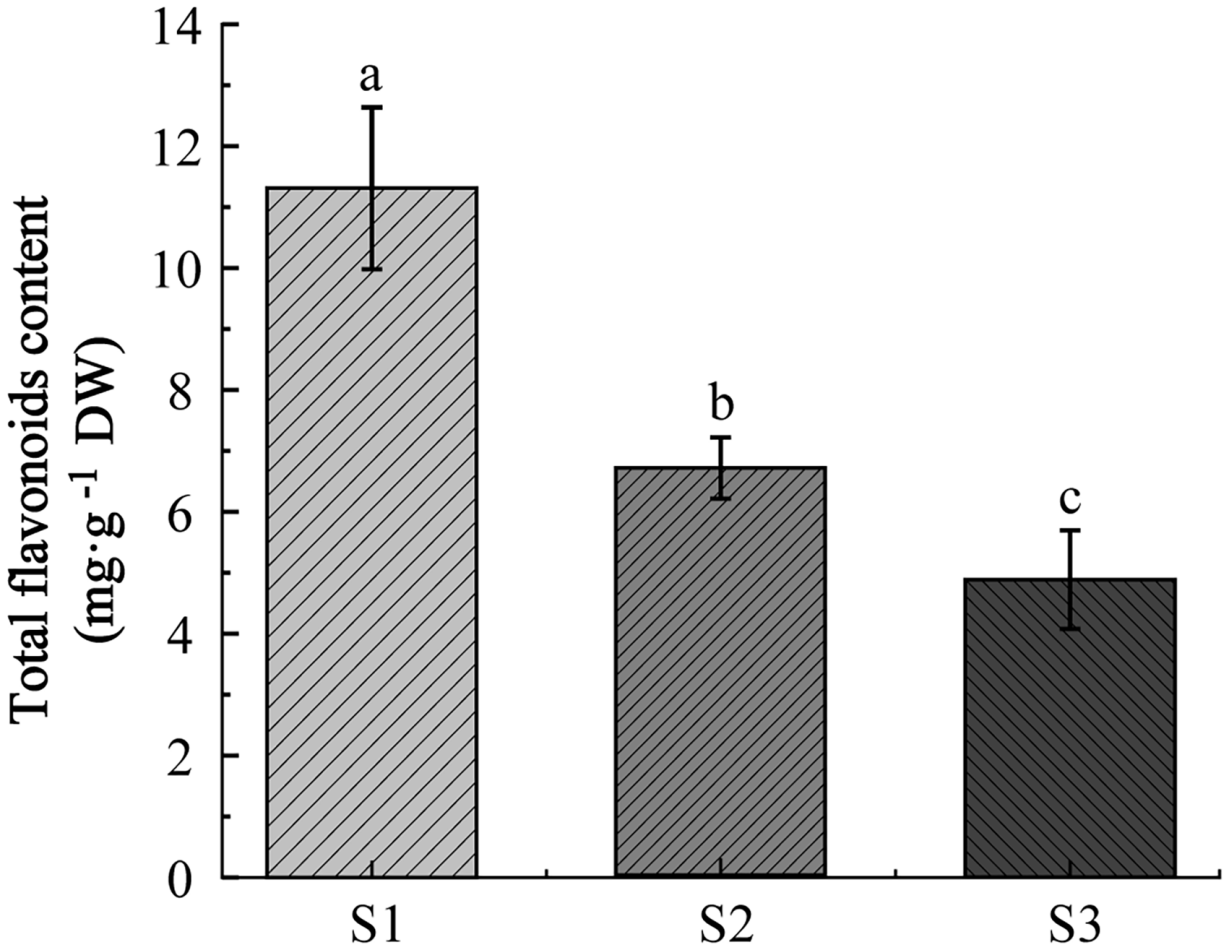
Determined the total content of flavonoids in chayote fruits at three storage stages (S1 ~ S3). Error bars represented ±SD. Different lower-case letters indicate a significant difference between group means (*p* < 0.05). S1, optimal harvest stage; S2, 15 days after storage; S3, 30 days after storage.

To explore the dynamic changes of flavonoids in chayote fruits during 1 month storage, the total content of flavonoids in chayote fruits at three different storage periods (S1, S2, and S3) was detected. The total content of flavonoids in S1 stage (11.31 mg. g^−1^, DW) was higher than those in S2 stage (6.68 mg. g^−1^, DW) and S3 stage (4.88 mg. g^−1^, DW), and the total content of flavonoids in S2 stage (6.68 mg. g^−1^, DW) was higher than that in S3 stages, indicating that the total content flavonoids was decreased in chayote during 1 month of storage (Figure 1).

**Figure 2:**
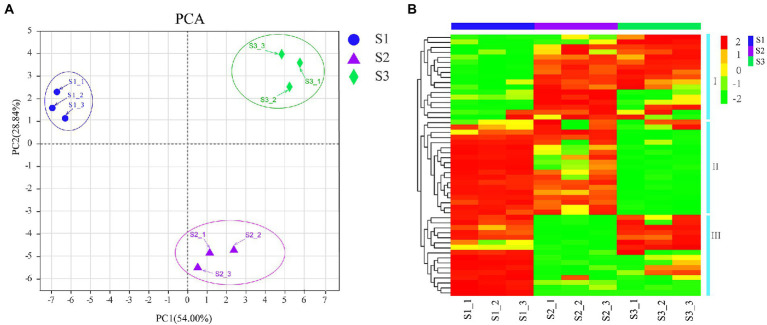
PCA and clustering heat map of flavonoid compounds. **(A)** PCA score plots; **(B)** Clustering heatmap were drawn with the relative contents of flavonoid compounds. Red and green colors represent the higher or lower content of flavonoid metabolites, respectively.

To further examine the dynamic changes of flavonoid compounds in chayote fruits during storage, untarget metabolite profiling was conducted through the UPLC-MS/MS system. A total of 57 flavonoid compounds, including 42 flavonoid glycosides, 3 O-methylated flavonoids, 2 flavans, 2 flavones, 1 isoflavonoid, 1 isoflavan, 3 isoflavonoid O-glycosides, 1 O-methylated isoflavonoid, 1 prenylated neoflavonoid, and 1 pyranoisoflavonoid, were identified in chayote fruits at three different storage periods (Figure 2; Table 1). Moreover, the principal component analyses (PCA) of metabolite accumulation levels in chayote fruits. Two principal components, PC1 (28.84%) and PC2 (54%), explained 82.84% of the variation in the metabolic profile (Figure 2A). The 18 samples were divided into three groups along PC1, indicating that the model was suitable. A weak correlation among the biological repetitions of the S3 stage and a clear separation among S1, S2, and S3 were observed based on PCA (Figure 2A). Visualization of the flavonoid profile was performed by hierarchical cluster analysis (HCA; Figure 2B). As shown in Figure 2B, the metabolite profiles were clustered into three clusters: clusters I, II, and III, and the results showed that the flavonoid compounds in chayote fruits were differentially accumulated distributed in the three periods (S1, S2, and S3) of chayote storage (Figure 2B). In cluster I, the flavonoid compounds in S2 stage of chayote storage was higher than that in S3 stage, and the flavonoids in S1 stage of chayote storage was not detected (Figure 2B). In cluster II, the flavonoid compounds in S1 stage was higher than that in S2 stage, and the content of flavonoids in S3 stage was relatively low (Figure 2B). In cluster III, the flavonoid compounds in S1 stage was significantly higher than that in S3 stage, and the content of flavonoids in S2 stage was relatively low (Figure 2B). In addition, the number of flavonoid compounds in S1 stage was higher than that in S2 stage, and the number of flavonoid compounds in S2 stage was higher than that in S3 stage (Figure 2B).

**Table 1:** Checklist of flavoniods and molecular features analyzed in this study.

Metab ID	Metab ID	Metabolite	Mass error	Subtype	S1	S2	S3
metab_8444	neg_614	Azaleatin 3-rutinoside	9.483114607	Flavonoid glycosides	2.14	2.12	2.11
metab_15484	neg_7654	Isopeonidin 3-rutinoside	−1.918581594	Flavonoid glycosides	3.41	3.21	2.43
metab_640	pos_641	Diosmin	9.483114607	Flavonoid glycosides	4.73	5.00	5.00
metab_7173	pos_7174	Hesperidin	9.483114607	Flavonoid glycosides	2.17	1.97	0.80
metab_10464	neg_2634	Negletein 6-[rhamnosyl-(1- > 2)-fucoside]	Mass Error	Flavonoid glycosides	2.20	1.95	1.57
metab_14725	neg_6895	Hesperetin 7-(2,6-dirhamnosylglucoside)	−0.680081424	Flavonoid glycosides	0.36	2.46	0.63
metab_648	pos_649	Glucoliquiritin apioside	9.483114607	Flavonoid glycosides	3.15	1.46	0.83
metab_6862	pos_6863	RHOIFOLIN	9.483114607	Flavonoid glycosides	3.69	3.60	3.62
metab_6944	pos_6945	Isovitexin	9.483114607	Flavonoid glycosides	2.25	2.23	2.70
metab_7064	pos_7065	3,8-Di-C-glucopyranosylapigenin	9.483114607	Flavonoid glycosides	3.32	2.81	2.83
metab_7120	pos_7121	Apigenin 7-[rhamnosyl-(1- > 2)-galacturonide]	9.483114607	Flavonoid glycosides	2.44	1.70	0.78
metab_2783	pos_2784	Diosmetin	9.483114607	O-methylated flavonoids	2.43	2.60	2.65
metab_6733	pos_6734	Kaempferol 3-alpha-D-glucoside	9.483114607	Flavonoid glycosides	0.78	1.59	2.02
metab_10472	neg_2642	Kaempferol 3-arabinofuranoside 7-rhamnofuranoside	Mass Error	Flavonoid glycosides	2.18	1.43	1.89
metab_10513	neg_2683	Kaempferol 3-O-glucosyl-(1- > 2)-rhamnoside	Mass Error	Flavonoid glycosides	3.03	2.55	2.63
metab_14970	neg_7140	KAEMPFEROL-7-O-GLUCOSIDE	−5.093366433	Flavonoid glycosides	0.86	2.14	2.42
metab_1205	pos_1206	Glycitein 7-O-glucuronide	0.003212067	Isoflavonoid O-glycosides	1.48	1.92	1.91
metab_1269	pos_1270	5,7-dihydroxy-2-phenyl-8-(3,4,5-trihydroxyoxan-2-yl)-4H-chromen-4-one	−0.943369701	Flavonoid glycosides	1.36	1.98	2.01
metab_2581	pos_2582	5,6,7-trihydroxy-8-(3-methylbut-2-en-1-yl)-4-phenyl-2H-chromen-2-one	9.483114607	Prenylated neoflavonoids	1.98	1.40	1.11
metab_2739	pos_2740	Ganoderic acid L	9.483114607	O-methylated isoflavonoids	1.17	1.86	1.47
metab_2750	pos_2751	Isorhamnetin 3-O-[b-L-rhamnofuranosyl-(1- > 6)-D-glucopyranoside]	9.483114607	Flavonoid glycosides	1.94	1.63	1.62
metab_5745	pos_5746	Lucidenic acid M	9.483114607	O-methylated flavonoids	1.74	1.33	0.46
metab_6431	pos_6432	5,4’-Dihydroxy-3,3′-dimethoxy-6:7-methylenedioxyflavone	9.483114607	O-methylated flavonoids	1.18	1.57	1.58
metab_6565	pos_6566	Naringin 6″-rhamnoside	9.483114607	Flavonoid glycosides	0.62	2.34	2.29
metab_6688	pos_6689	Cadabicine methyl ether	9.483114607	Flavonoid glycosides	1.38	0.81	0.84
metab_7028	pos_7029	6″-p-Coumaroylprunin	9.483114607	Flavonoid glycosides	1.68	0.93	2.05
metab_7120	pos_7121	Apigenin 7-[rhamnosyl-(1- > 2)-galacturonide]	9.483114607	Flavonoid glycosides	2.44	1.70	0.78
metab_7267	pos_7268	3,5,6-Trihydroxy-3′,4′,7-trimethoxyflavone 3-glucuronide	9.483114607	Flavonoid glycosides	1.93	0.10	0.33
metab_7269	pos_7270	4’-O-Methylkanzonol W	9.483114607	Pyranoisoflavonoids	2.57	2.14	0.93
metab_7512	pos_7513	Lathycarpin	9.483114607	Furanoisoflavonoids	2.42	2.25	2.62
metab_7519	pos_7520	Tricin 7-glucuronoside	9.483114607	Flavonoid glycosides	2.17	2.31	2.43
metab_8428	neg_598	Vestitone 7-glucoside	9.483114607	Isoflavonoid O-glycosides	2.14	1.66	1.35
metab_9622	neg_1792	Aspalathin	9.483114607	Flavones	1.77	2.38	1.85
metab_10237	neg_2407	(2R,3R)-3,3′,4′,7-Tetrahydroxyflavanone 7-O-alpha-L-Rhamnopyranoside	Mass Error	Flavonoid glycosides	2.32	2.22	2.32
metab_10380	neg_2550	6”-O-Malonylnaringin	Mass Error	Flavonoid glycosides	1.52	0.57	1.29
metab_10464	neg_2634	Negletein 6-[rhamnosyl-(1- > 2)-fucoside]	Mass Error	Flavonoid glycosides	2.20	1.95	1.57
metab_10481	neg_2651	Peonidin 3-sophoroside 5-glucoside	Mass Error	Flavonoid glycosides	1.32	1.25	1.46
metab_10538	neg_2708	4′-Methylliquiritigenin 7-rhamnoside	Mass Error	Flavonoid glycosides	1.73	1.80	2.50
metab_10647	neg_2817	Astilbin	−0.891811124	Flavonoid glycosides	1.15	1.40	0.62
metab_10685	neg_2855	Albanin H	−0.891811124	Flavones	1.32	2.42	1.38
metab_10774	neg_2944	Heteroflavanone C	−0.891811124	Flavans	0.92	1.46	1.02
metab_10826	neg_2996	Kanzonol G	−0.891811124	Isoflavans	1.71	1.21	0.60
metab_14140	neg_6310	6”-Caffeoylhyperin	−1.084405102	Flavonoid glycosides	1.78	2.21	2.16
metab_14662	neg_6832	Isorhamnetin 3-(6″-malonylglucoside)	−0.680081424	Flavonoid glycosides	0.60	1.71	1.67
metab_14666	neg_6836	6″-Malonylcosmosiin	−0.680081424	Flavonoid glycosides	2.66	2.96	2.92
metab_14725	neg_6895	Hesperetin 7-(2,6-dirhamnosylglucoside)	−0.680081424	Flavonoid glycosides	0.36	2.46	0.63
metab_15089	neg_7259	Lupiwighteone hydrate 7-glucoside	−1.892106637	Isoflavonoid O-glycosides	1.37	0.19	1.17
metab_15145	neg_7315	Demethyloleuropein	−1.884779898	Flavonoid glycosides	2.58	2.22	2.62
metab_15176	neg_7346	3′,5,6-Trihydroxy-3,4′,7,8-tetramethoxyflavone 3-glucoside	−1.884779898	Flavonoid glycosides	3.00	2.63	3.08
metab_9834	neg_2004	4 beta-(2-Aminoethylthio)epicatechin 3-gallate	9.483114607	Flavonoid glycosides	2.80	2.95	3.00
metab_15346	neg_7516	Isovitexin 2”-O-glucoside	3.827889426	Flavonoid glycosides	3.76	3.28	3.05
metab_15368	neg_7538	Taxifolin 3-arabinoside	8.672078042	Flavonoid glycosides	2.01	1.01	2.55
metab_15535	neg_7705	Aromadendrin 3,7-diglucoside	4.041258207	Flavonoid glycosides	3.54	3.19	2.85
metab_15591	neg_7761	Pelargonidin 3-rhamnoside	7.433091171	Flavonoid glycosides	1.39	1.11	0.39
metab_15297	neg_7467	Pelargonidin 3-sophoroside	−0.615696317	Flavonoid glycosides	1.58	0.79	0.49
metab_14871	neg_7041	Kaempferol 3-rhamnoside 7-xyloside	−0.680081424	Flavonoid glycosides	2.95	2.37	2.56
metab_15311	neg_7481	6″-(4-Carboxy-3-hydroxy-3-methylbutanoyl) hyperin	4.284782139	Flavonoid glycosides	1.43	1.30	2.07

### 3.2. Different concentration of flavonoids in chayote fruits during storage

**Figure 3:**
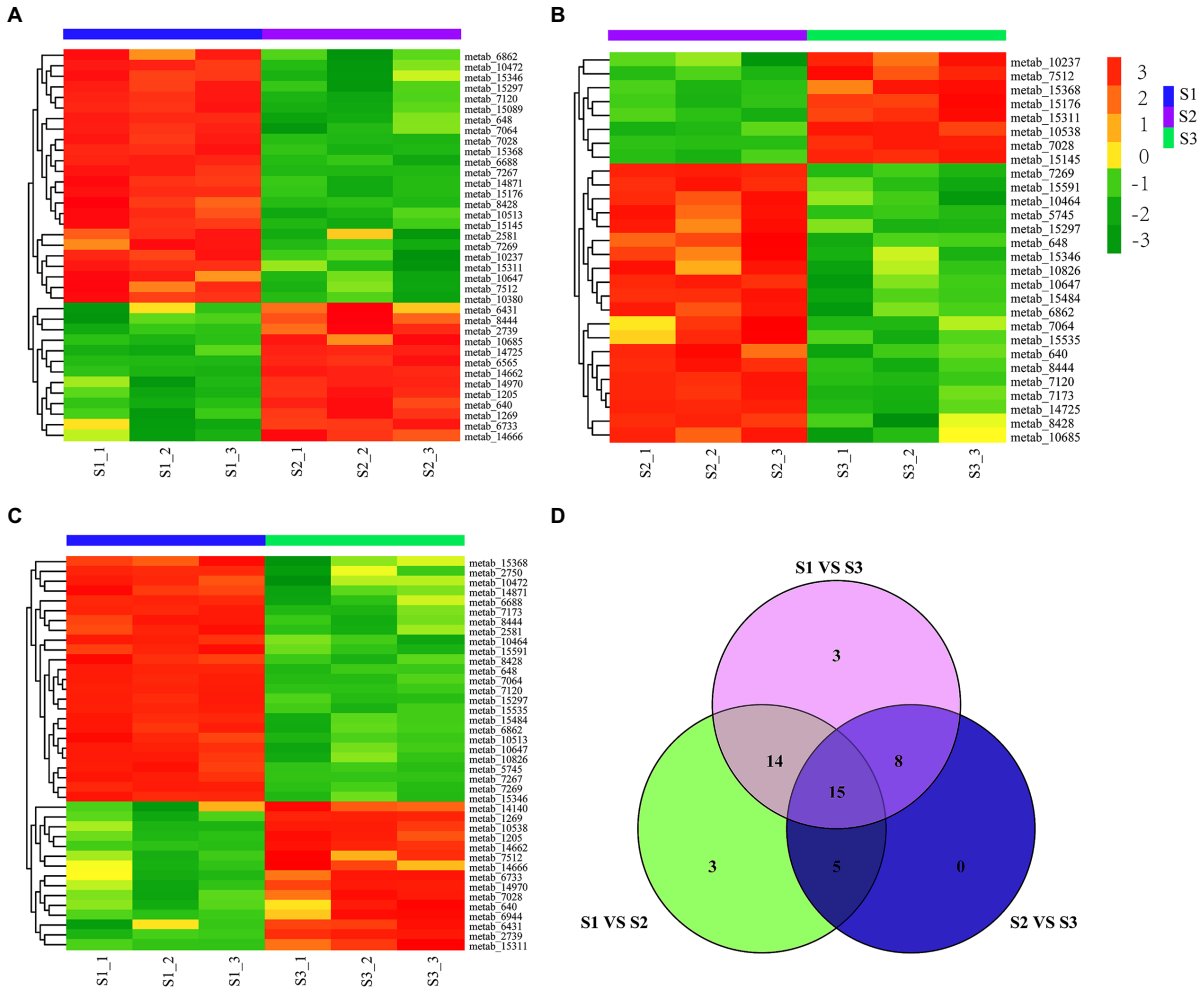
Differential flavonoid metabolites in chayote fruits during storage. **(A)** Differential in S1 vs. S2. **(B)** Differential flavonoid metabolites in S2 vs. S3. **(C)** Differential flavonoid metabolites in S1 vs. S3. **(D)** Venn diagram of differential flavonoid metabolites in different comparison groups. The red and green color in the plot represents higher and lower metabolite contents, respectively. S1, optimal harvest stage; S2, 15 days after storage; S3, 30 days after storage.

To further compare the differential accumulation of flavonoids in chayote fruits at three different storage stages, the pairwise comparison analysis of flavonoids in S1, S2, and S3 stages of chayote storage (Figure 3). The comparative analysis between S1 and S2 showed that 24 and 11 flavonoid compounds were increased and decreased in S1 vs. S2, respectively (Figure 3A). The increased flavonoid compounds contained 20 flavonoid glycosides, 1 prenylated neoflavonoids, 1 pyranoisoflavonoids, 1 isoflavonoid O-glycosides and 1 furanoisoflavonoids, and the decreased flavonoid compounds included 9 flavonoid glycosides, 1 Isoflavonoid O-glycosides, 1 O-methylated flavonoids, 1 O-methylated isoflavonoids and 1 flavones (Supplementary Table S2). The differential flavonoid compounds between S1 and S3 showed that 25 flavonoid compounds (20 flavonoid glycosides, 1 isoflavans, 1 isoflavonoid O-glycoside, 1 O-methylated flavonoid, 1 prenylated neoflavonoid, and 1 pyranoisoflavonoid) and 15 flavonoid compounds (11 flavonoid glycosides, 1 furanoisoflavonoids, 1 isoflavonoid O-glycosides, 1 O-methylated flavonoids, and 1 O-methylated isoflavonoids) were upregualted and downregulated in S1 vs. S3, respectively (Figure 3B; Supplementary Table S3). The pairwise comparison analysis between S2 and S3 showed that 20 flavonoid compounds (15 flavonoid glycosides, 1 flavone, 1 isoflavans, 1 isoflavonoid O-glycoside, 1 O-methylated flavonoid, and 1 pyranoisoflavonoid) and 8 flavonoid compounds (7 flavonoid glycosides, 1 furanoisoflavonoids) were increased and decreased in S2 vs. S3, respectively (Figure 3C; Supplementary Table S4). Notably, 20, 23, and 29 differential flavonoid metabolites were both present in S1 vs. S2 and S2 vs. S3, S2 vs. S3 and S1 vs. S3, and S1 vs. S2 and S1 vs. S3, respectively. Moreover, 15 flavonoid compounds, including 12 flavonoid glycosides, 1 furanoisoflavonoids, 1 isoflavonoid O-glycosides, and 1 pyranoisoflavonoid, were present in all three comparison groups (Figure 3D; Supplementary Table S5). In addition, 69.2% (27), 79.5% (31), and 53.8% (21) of 42 flavonoid glycosides were differentially accumulated in S1 vs. S2, S1 vs.S3, and S2 vs.S3, respectively, suggesting that the reduction of flavonoid glycosides resulted in a decrease in flavonoid content during chayote fruit storage at room temperature.

### 3.3. Transcriptome analysis of chayote fruits during storage

To clarify the molecular mechanisms involved in flavonoid synthesis in chayote fruits during storage, we constructed nine cDNA libraries from the same samples used for metabolic analysis and performed RNA-seq analysis. In this study, we totally achieved 470,430,862 raw reads and 465,666,530 clean reads. The Q30 percentage and average GC content were above over 93 and 46% for all libraries, respectively. Moreover, 84.62 to 86.76% of clean reads in each library were assembled to the reference genome for each library. A total of 57,814 unigenes were identified, with 26,742, 27,925, 27,534, 28,559, 29,222, 28,172, 27,956, 28,826, and 28,201 unigenes in each library (Table 2). This result indicated that the high quality of RNA-seq data can be suitable for subsequent analyses.

**Table 2:** Summary statistics of RNA-seq results in chayote fruits at three different storage stages.

Sample	Raw reads	Raw bases	Clean reads	Clean bases	Q30 (%)	GC content (%)	Mapped reads	Unigene
S1_1	46,660,132	7,045,679,932	46,123,892	6,818,399,529	94.3	46.2	39,807,196	26,742
S1_2	58,295,122	8,802,563,422	57,640,298	8,504,638,824	94.3	46.2	50,009,600	27,925
S1_3	44,777,442	6,761,393,742	44,282,274	6,532,048,804	94.4	46.0	37,930,180	27,534
S2_1	67,316,602	10,164,806,902	66,557,990	9,815,349,303	94.2	46.1	56,945,070	28,559
S2_2	47,413,054	7,159,371,154	46,847,124	6,905,042,899	93.8	45.9	39,767,366	29,222
S2_3	53,269,952	8,043,762,752	52,673,966	7,829,079,828	93.6	45.9	44,573,538	28,172
S2_1	51,124,548	7,719,806,748	50,685,390	7,549,027,732	95.8	45.8	43,317,136	27,956
S2_2	45,091,272	6,808,782,072	44,768,820	6,656,801,371	95.8	45.7	38,182,204	28,826
S2_3	56,482,738	8,528,893,438	56,086,776	8,369,322,866	95.7	45.9	47,621,380	28,201

**Figure 4:**
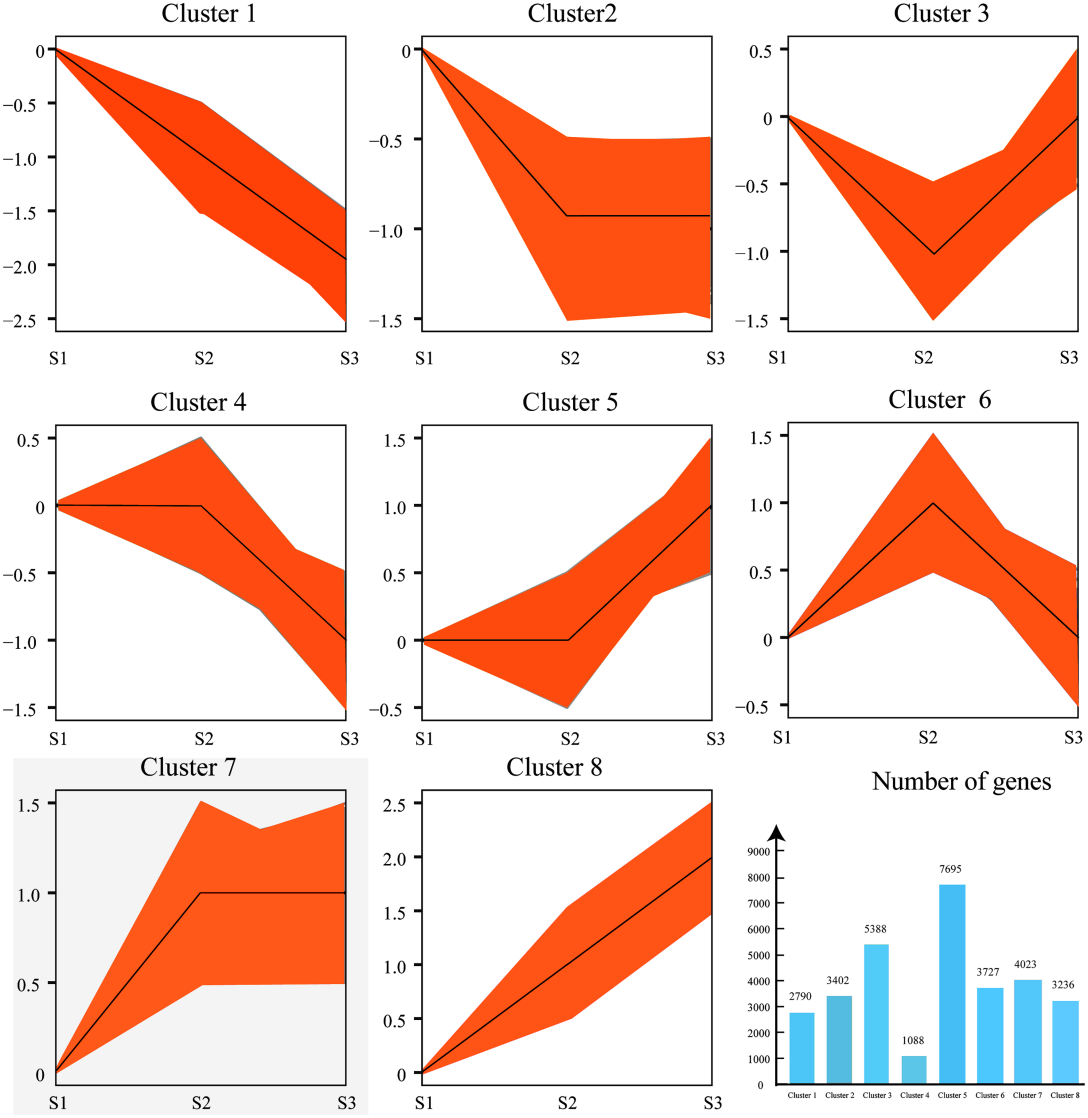
Clustering of gene expression profiles from chayote fruits during storage. The k-means method was used to perform the cluster analysis of gene expression profiles. The number of DEGs in each cluster. The *X*-axis and *Y*-axis represent the different samples and normalized FPKM of DEGs.

**Figure 5:**
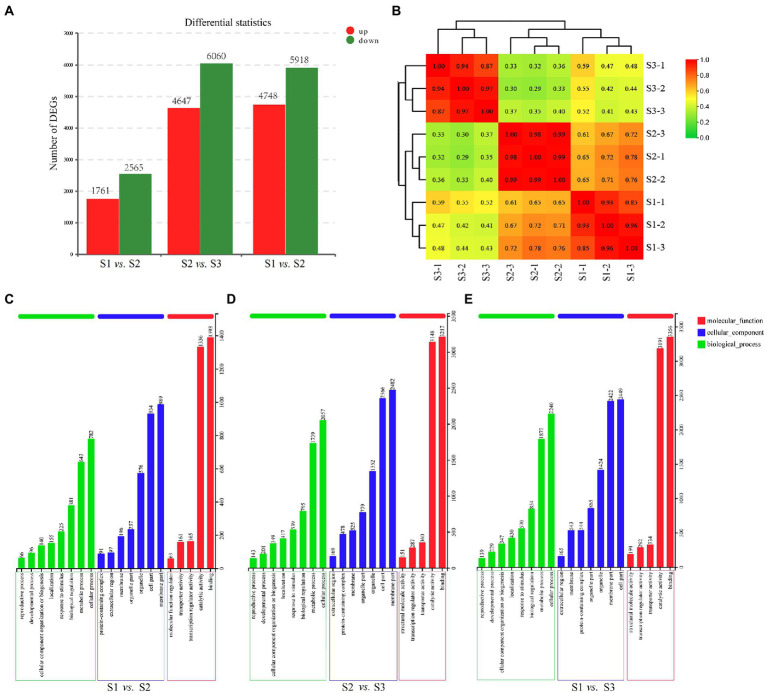
Transcriptome analysis for the fruits at the different developmental stages. **(A)** The number of DEGs in different comparison groups. **(B)** Relationship of RNA-seq. **(C)** GO enrichment analysis of DEGs in S1 vs. S2. **(D)** GO enrichment analysis of DEGs in S2 vs. S3. **(E)** GO enrichment analysis of DEGs in S1 vs. S3.

In order to explore the DEGs in chayote fruits during storage, we performed the correlation coefficients and gene expression clustering among the samples from different biological replicates. The k-means method was used to group 57,814 unigenes into eight different clusters, suggesting that the DEGs were differentially expressed in chayote fruit samples at different storage stages (Figure 4). Gene expression correlations among different biological replicates were higher than 0.85 (Figure 5B), implying that biological replicates were completely distinguishable, and identification of DEGs has been performed based on the RNA-seq data. Thus, 4,326 (1,761 up-and 2,565 down-regulated), 10,707 (4,647 up-and 6,060 down-regulated), and 10,666 (4,748 up-and 5,918 down-regulated) DEGs were found in S1 vs. S2, S2 vs. S3, and S1 vs. S3, respectively (Figure 5A).

To investigate the function of the DEGs, Blast-GO was used to perform GO-term enrichment analysis. 8,726 DEGs (S1 vs. S2), 21,554 DEGs (S2 vs. S3), and 22,460 DEGs (S1 vs. S3), were classified into 20 function categories including 8 biological process (BP), 7 cellular component (CC), and 5 molecular function (MF; Figures 5C–E). In biological processes, the most abundant term is the cellular process, followed by the metabolic process. For the cellular component categories, the greatest abundant terms are the cell part and the membrane part. Under the molecular component categories, binding and catalytic activity were the most representative terms.

**Figure 6:**
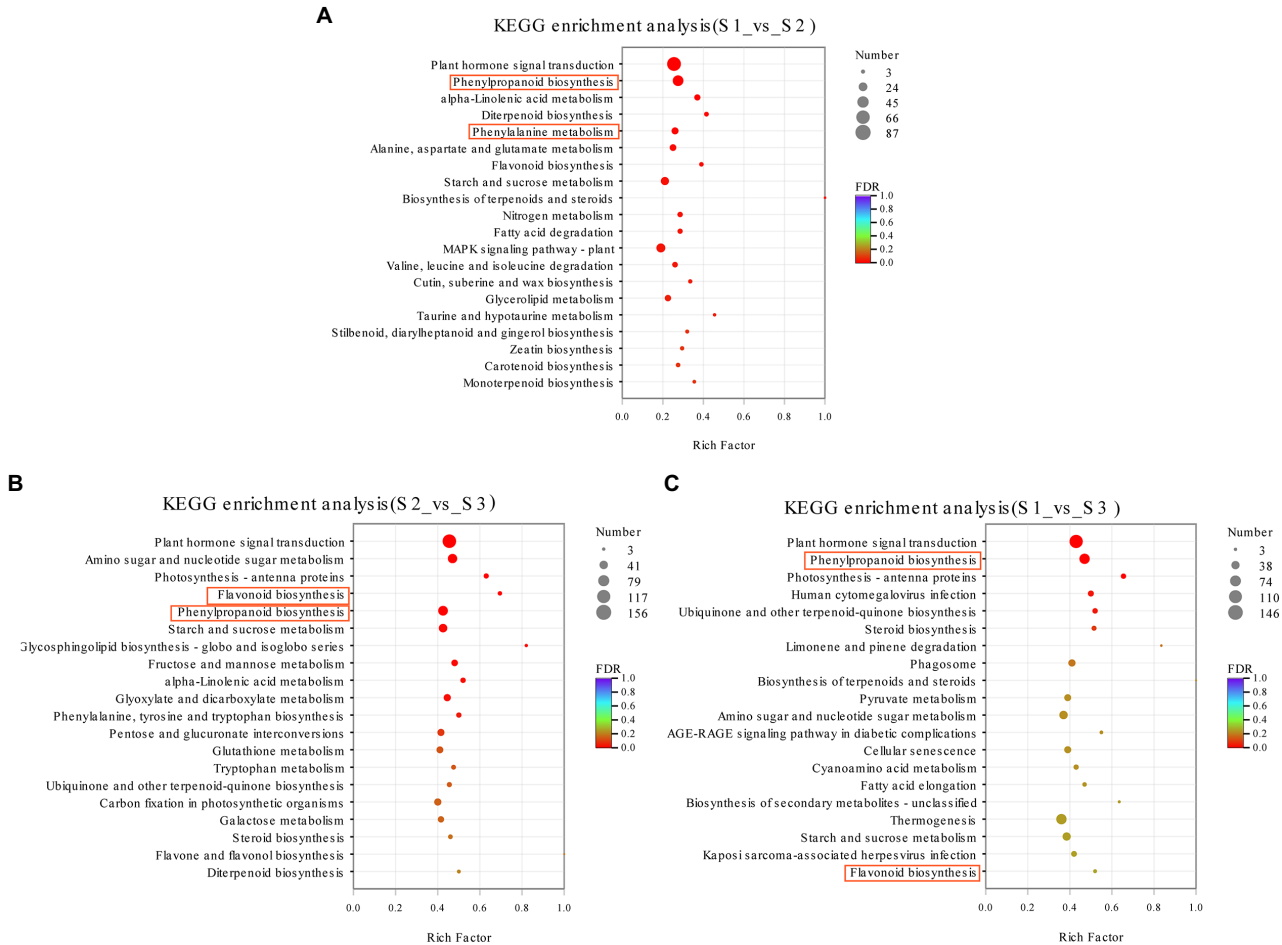
Significantly enriched KEGG pathway of all DEGs. **(A)** Pathway enrichment analysis of DEGs in S1 vs. S2 using the KEGG repository. **(B)** Pathway enrichment analysis of DEGs in S2 vs. S3 using the KEGG repository. **(C)** Pathway enrichment analysis of DEGs in S1 vs. S3 using the KEGG repository (*p* < 0.05).

The metabolic pathways of the DEGs were identified using the KEGG enrichment analysis. In present study, 1,091 (S1 vs. S2), 2,877 (S2 vs. S3), and 2,863 (S1 vs. S3) DEGs were assigned to 126, 140, and 140 KEGG pathways, respectively (Figure 6). Among them, 16, 11, and 5 significantly enriched pathways were identified with a *p* value of <0.05 (Figure 6). Notablely, the most highly enriched pathways in S1 vs. S2 and S1 vs. S3 were phytohormones signal transduction (ko04075) and phenylpropanoid synthesis (ko00940; Figures 6A, B). In addition, the significantly enriched pathways from S1 vs. S2, S2 vs. S3, and S1 vs. S3 were further grouped into 5 categories: cellular processes, environmental information processing, genetic information processing, metabolism and organismal systems (Figures 6A–C). Among these categories, the metabolism category contained the most abundant pathways. The top three most abundant KEGG pathways of DEGs from S1 vs. S2 in the metabolism category were involved in phytohormones signal transduction pathway (ko04075, 87 genes), phenylpropanoid synthesis pathway (ko00940, 52 genes), and MAPK signaling pathway (ko04016, 36 genes; Figure 6A; Supplementary Table S6). In S2 vs. S3, DEGs were most highly enriched in phytohormones signal transduction pathway (ko04075, 156 genes), phenylpropanoid synthesis pathway (ko00940, 80 genes), and amino sugar and nucleotide sugar metabolism pathway (ko00520, 74 genes; Figure 6B; Supplementary Table S6). In pairwise comparison of S1 vs. S3, phytohormones signal transduction (ko04075, 146 genes) and phenylpropanoid synthesis (ko00940, 88 genes) were the most highly enriched pathways (Figure 6C; Supplementary Table S6).

**Figure 7:**
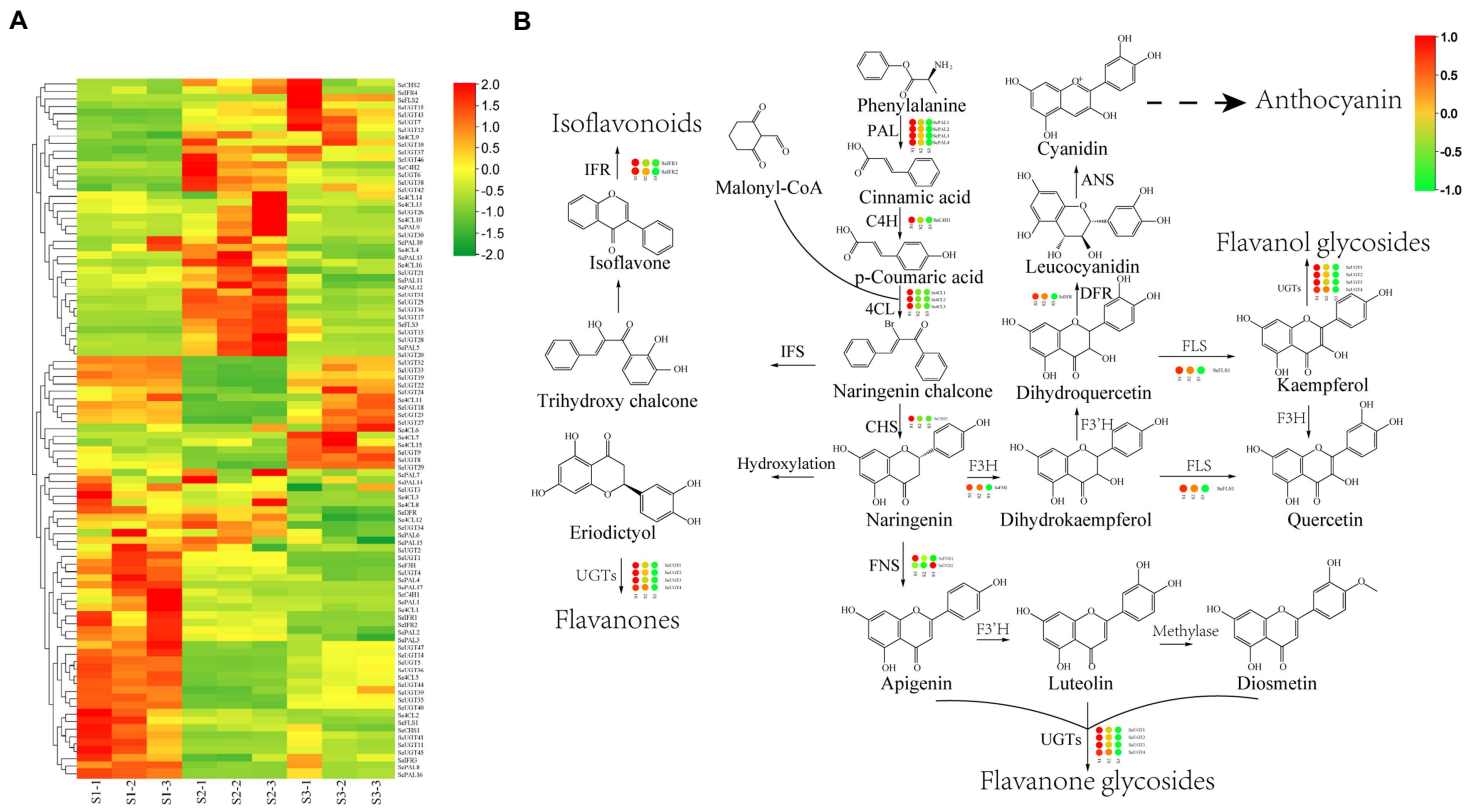
Identification of DEGs encoding the key enzymes associated with flavonoids synthesis**. (A)** Heatmap showing the expression of DEGs in chayote fruits at three different storage stages. **(B)** Expression profiles of DEGs involved in the flavonoid synthesis pathway. Upregulated (red) and downregulated (green) genes are indicated.

DEGs encoding key enzymes involved in flavonoid synthesis were detected based on the gene functional annotation and KEGG enrichment analysis. In present study, a total of 95 DEGs, including 16 *PAL* genes, two *C4H* genes, 16 *4CL* genes, two *CHS* gene, one *DFR* gene, one *F3H* gene, two *FNSI* genes, three *FLS* genes, five *IFR* genes and 47 *UGTs* were found in S1 vs. S2 and S2 vs. S3 (Figure 7A; Supplementary Table S7). Among them, 4 *PAL*, 1 *C4H*, 3 *4CL*, 1 *CHS*, 1 *DFR*, 1 *F3H*, 1 *FNSI*, 1 *FLS*, 2 *IFR*, and 4 *UGT* were significantly increased in S1 vs. S2 and S2 vs. S3 (Figure 7B; Supplementary Table S8). In addition, a total of 47 differentially expressed *UGT* genes were found in S1 vs. S2 and S2 vs. S3 (Figure 7A; Supplementary Table S7). Among them, four *UGT* genes were significantly increased in S1 vs. S2 and S2 vs. S3, respectively (Figure 7B).

Previous studies demonstrated that MYB and bHLH TFs played important roles in regulating flavonoid synthesis (35, 36). In addition, the WRKY, bZIP, and Dof TFs had been reported to be involved in the regulation of flavonoid synthesis and metabolism (37–39). Thus, these five class TFs, including 45 MYB, 33 bHLH, 25 WRKY, 20 bZIP, and 22 Dof TFs, were identified and differentially expressed in at least one comparison group (Supplementary Table S9). Among them, 12 MYB, 8 bHLH, 1WRKY, 6 bZIP, and 2 Dof TFs were significantly increased in S1 vs. S2 and S2 vs. S3, which were consistent with the expression patterns of structural genes involved in flavonoid synthesis (Supplementary Table S10). In contrast, the expression pattern of 12 MYB, 6 bHLH, 9 WRKY, 5 bZIP, and 6 Dof TFs was opposite to that of structural genes involved in flavonoid synthesis, which were down-regulated in S1 vs. S2 and S2 vs. S3 (Supplementary Table S8).

### 3.4. Verification of DEGs involved in flavonoid synthesis using qRT-PCR

**Figure 8:**
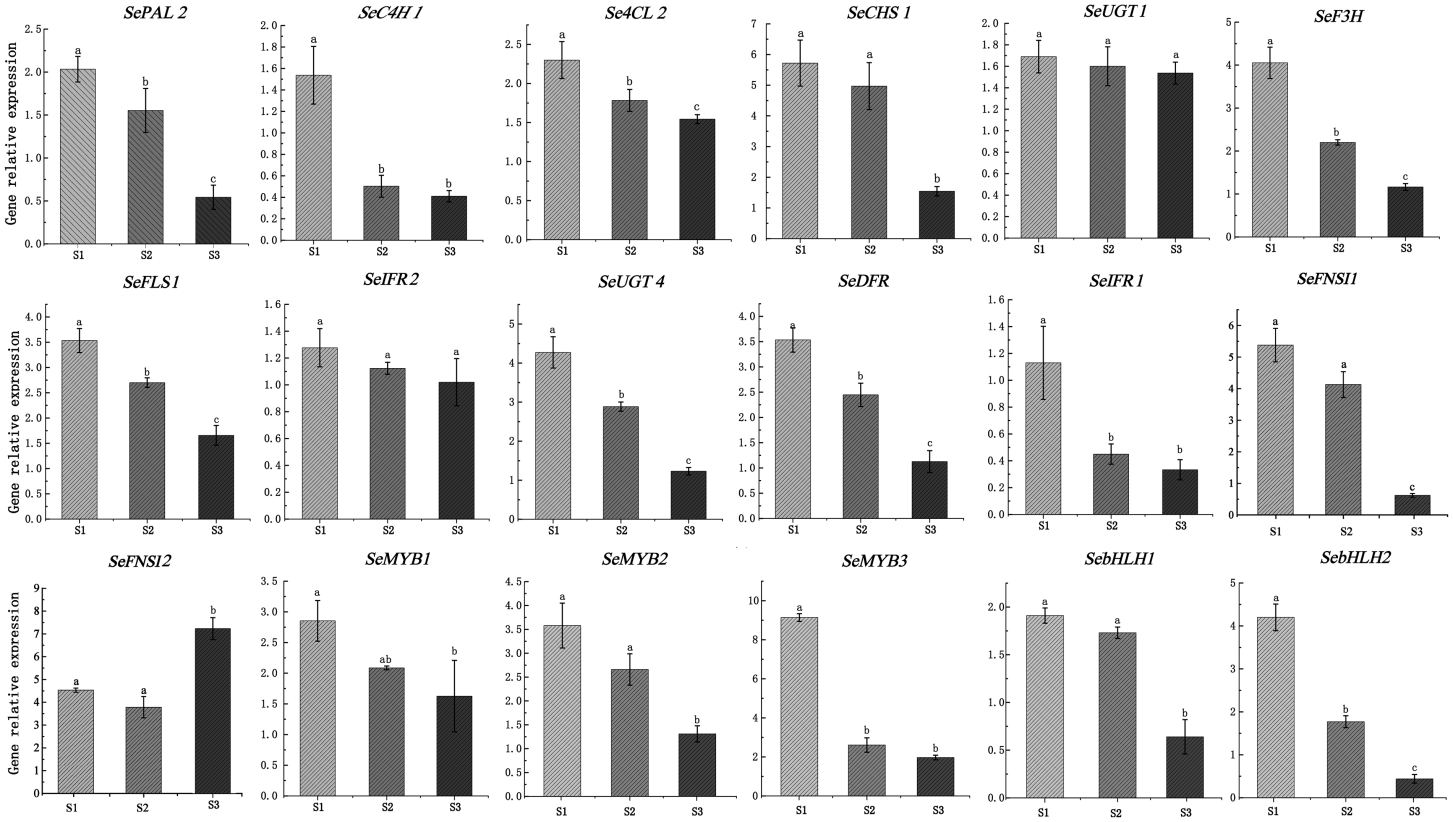
Validation of expression pattern of 18 DEGs associated with flavonoid synthesis using qRT-PCR. Relative expression levels of qRT-PCR were calculated using actin 7 as a standard. Three biological replicates and three technical replicates were obtained for each data point. Different lower-case letters indicate a significant difference among the different stages (*p* < 0.05). PAL, phenylalanine ammonia-lyase; C4H, cinnamate-4-hydroxylase; 4CL, 4-coumarate-CoA ligase; F3H, flavanone 3-hydroxylase; FLS, flavonol synthase; IFR, isoflavone reductase; DFR, dihydroflavonol 4-reductase; FNSI, flavone synthase I; UGT, UDP-glycosyltransferase.

To further verify the accuracy of RNA-seq data, a total of 18 DEGs, including 13 structural genes (1 *PAL*, 1 *C4H*, 1 *4CL*, 1 *CHS*, 1 *F3H*, 2 *FNSI*, 1 *FLS*, 1 *IFR*, 2 *UGT*, 1 *DFR*, 1 *IFR*) and 5 TFs (3 MYB and 2 bHLH) were used to explore the expression patterms in chayote fruits at three different storage stages (S1, S2, and S3) using qRT-PCR. As shown in Figure 8, the expression pattern of most selected DEGs was consistent with the results of RNA-seq data (Supplementary Table S10).

### 3.5. Correlation analysis between DEGs and DEMs

**Figure 9:**
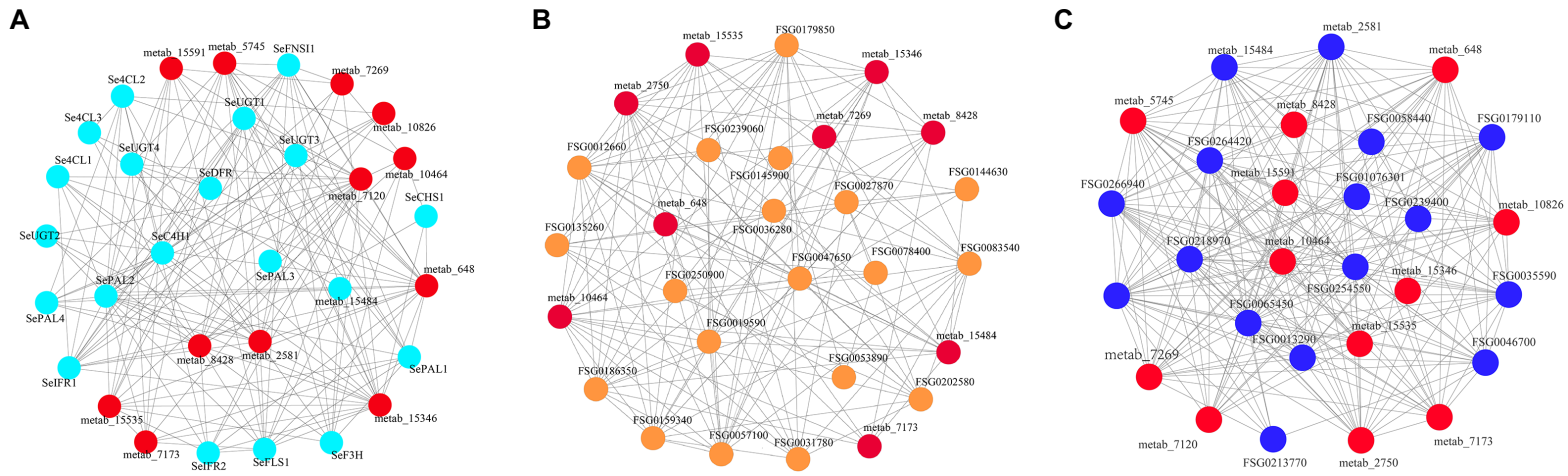
Connection network between DEGs and TFs associated with flavonoid synthesis and DEMs in chayote fruits during storage. **(A)** Correlation analysis between structural genes of flavonoid synthesis and flavonoids; **(B)** Correlation analysis between MYB TFs and flavonoids; **(C)** Correlation analysis between bHLH TFs and flavonoids. The red ellipsoid represents the DEMs. The light blue, yellow, blue, pink and green ellipsoid represent structure genes, MYB, and bHLH TFs, respectively.

To explore the complexity of regulatory networks that control flavonoid synthesis in chayote fruits during storage, the correlation analysis between quantitative changes of flavonoid-synthesis-related DEGs and DEMs in chayote fruits at three different storage stages were performed (Figure 9). In this study, 17 structural genes were highly correlated with 13 flavonoid compounds (*r* > 0.8, Figure 9A; Supplementary Table S11). Eighteen structural genes, including 3 *PAL*, 1 *C4H*, 3 *4CL*, 1 *CHS*, 1 *DFR*, 1 *F3H*, 1 *FNSI*, 1 *FLS*, 2 *IFR* and 4 *UGT*, were identified as key structural genes involved in the flavonoid synthesis. The 13 flavonoids including 8 flavonoid glycosides, 1 isoflavans, 1 isoflavonoid O-glycosides, 1 O-methylated flavonoids,1 prenylated neoflavonoids, and 1 pyranoisoflavonoids. Moreover, a specific correlation analysis between the identified MYB and bHLH and DEMs was also conducted (Figures 9B, C). Our results showed that 21 MYB and 12 bHLH TFs were extremely correlated with 8 flavonoid compounds (*r* > 0.8; Figures 9B, C; Supplementary Table S12), respectively. Among them, one R2R3-MYB transcription factor, FSG0057100, the orthologous gene of *Arabidopsis* AtMYB12, belong to the R2R3-MYB TFs, which was involved in accumulating of plant flavonol glycoside (40).

### 3.6. The effect of penylalanine on fruits quality during storage

**Figure 10:**
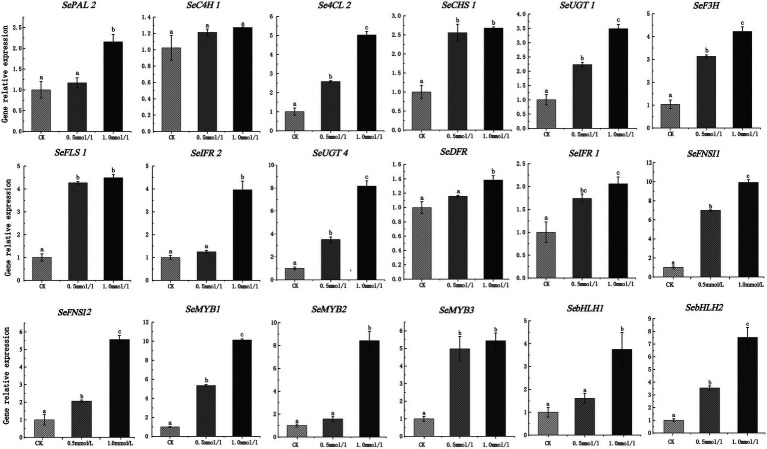
Expression profiles of 18 DEGs involved in flavonoid biosynthesis after phenylalanine treatment. The expression levels of DEGs relative to *actin7*, measured by qRT-PCR. Three biological replicates and three technical replicates were obtained for each data point. Lower case letters (a, b, c) above the error bars represent significant difference between the treated and untreated (0 g/l) samples (*p* < 0.05).

Phenylalanine is an aromatic amino acid that enters the phenylpropane pathway as the initial substrate, which is the precursor of flavonoids that improve the quality of crops (41). In this study, we treated the chayote fruits with different concentrations of phenylalanine, and the effect of phenylalanine on the flavonoid contents were explored. Our results showed that the total content of flavonoids in chayote fruits treated with 0.5 mM and 1 mM of phenylalanine were 1.27 times and 1.61 times that of the control group, respectively (Supplementary Figure S1). Moreover, the expression patterns of 18 DEGs in chayote fruits treated with phenylalanine were investigated using qRT-PCR. The expression level of all genes was significantly increased after treatment with different concentrations of phenylalanine compared to control group (Figure 10; Supplementary Table S13). The expression level of most structural genes in chayote fruits treated with 0.5 and 1 mM of phenylalanine were increased by 1.17 ~ 6.98 times and 1.25 ~ 9.92 times in comparison with control group. Moreover, the expression level of MYB TFs in chayote fruits treated with 0.5 and 1 mM of phenylalanine were increased by 1.38 ~ 5.35 times and 5.44 ~ 10.12 times in comparison with control group, respectively. In addition, the expression level of SebHLH1 and SebHLH2 in chayote fruits treated with 0.5 mM of phenylalanine was increased by 1.61 and 1.54 times in comparsion with control group, respectively, which were also increased by 3.75 and 7.52 times in chayote fruits treated with 1 mM of phenylalanine. These results suggested that exogenous phenylalanine applications could promote the accumulation of flavonoids by increasing key genes involved in flavonoid biosynthesis.

## 4. Discussion

### 4.1. Systematically detect the composition and content of flavonoid compounds In chayote fruits during storage

All organs of chayote plant are edible such as roots, shoots, leaves, and fruits, which was commonly used for medicinal and culinary purposes due to the rich minerals, dietary fibers, proteins, vitamins, polysaccharides, and flavonoids (42). Flavonoids are one of the major plant metabolites found throughout the plant kingdom, especially in fruits and vegetables. Fruits of chayote is rich in a variety of nutrients and bioactive compounds, such as polyphenols and flavonoids (43). The total phenolic contents of rowanberry fruits and banana fruits stored at room temperature for 20 day and 2 days were significantly decreased (19, 20). Aung et al. (44) reported that the quality of chayote (fresh weight) was affected by postharvest storage temperature and film wrap. Wang et al. (20) found that the total content of flavonoids in fruits of *Cerasus Humilis* kept decreasing with storage time. However, the effect of storage time on the total content and the changes in the components of flavonoids in chayote fruits was still unclear. Thus, the dynamic change of total flavonoid content in chayote fruits during storage was investigated, and found that the total flavonoid content was decreased in chayote fruits during 1 month storage (Figure 1), which was consistent with the previous studies (19–21).

Phenylalanine is an aromatic amino acid that enters the phenylpropane pathway as the initial substrate, which is the precursor of flavonoids that improve the quality of crops (41). Previous studies have revealed that exogenous application of phenylalanine can increase the expression level of flavonoid biosynthesis-related genes. Md-Mustafa et al. (45) showed that exogenous phenylalanine increased the expression of 2 *PAL*, 3 *4CL*, and 1 *CHS,* and the addition of phenylalanine (1 mM) increased the folate content up to 2.0-fold. Our results showed that the total content of flavonoids in chayote fruits treated with phenylalanine solutions (0.5 and 1 mM) was 1.27–1.61 times that of the control, and some flavonoid biosynthesis-related genes were significantly increased in chayote fruits treated with phenylalanine. These results suggested that phenylalanine increased the total flavonoid content in chayote fruits during storage by upregulated the expression of flavonoid biosynthesis-related genes. Phenylalanine can be used in fruit or vegetable storage, promote flavonoid biosynthesis, improve plant resistance to biotic and abiotic stresses, and increase plant quality.

### 4.2. Metabolic profiling and transcriptome analysis of chayote fruits during storage

Combined transcriptome analysis and metabolic profiling were widely used to explore the flavonoid metabolites and the corresponding molecular mechanism of flavonoid synthesis in many plants, such as tea, peach, *Physalis angulata*, and Mulberry (46–49). In this study, the flavonoid metabolites and corresponding molecular mechanism of flavonoid biosynthesis in chayote fruits during storage were explored using the combined transcriptome and metabolome profiling. A total of 57 flavonoid compounds were identified in chayote fruits during storage using UPLC-MS/MS. The number of flavonoid compounds in chayote fruits was higher than that in fruit peel of melon (40 differential flavonoids) (16), and less than that in cucumber yellow peel (165 differential flavonoids) (50) and in citrus fruit (117 differential flavonoids) (51). Moreover, 42 (72%) of the 57 flavonoid compounds belong to flavonoid glycosides, which was the main reason for the decrease of the total content of flavonoids (21, 52). Previous study showed that flavonoid glycosides are the main form of flavonoids in plants (53). In addition, flavonoid glycosides played important roles in response to abiotic stress (drought, cold, UV) (50, 54). Our results showed that the content of flavonoid glycosides in chayote fruits was decreased with storage time due to the loss of water (19, 20), which was consistent with previous study (53). Isoflavonoids were predominantly synthesized in leguminous plants, which played important roles in reducing osteoporosis and cardiovascular diseases, preventing cancer, and treating menopause symptoms (55–59). Moreover, six isoflavonoids and their derivatives were detected in chayote fruits for the first time, suggesting that the chayote fruit is new sources of isoflavonoids for human health.

### 4.3. Structure genes of phenylpropanoid pathway in plants

The phenylpropanoid pathway is responsible for the biosynthesis of numerous secondary metabolites such as phenylpropenes, anthocyanins, stilbenoids, and flavonoids, which regulated the plant growth and environmental response (60–62). The complex molecular mechanism of flavonoid synthesis was extensively studied in many plants (63–65). However, the molecular regulatory mechanism of flavonoid biosynthesis has not been systematically investigated in chayote fruits during storage. In this study, 95 significant DEGs involved in flavonoid biosynthesis (e.g., *PAL*, *C4H*, *4CL*, *CHS*, *CHI*, *F3'H*, *F3'5'H*, *FLS*, *FNSI*, and *UGT*) were identified based on the functional annotation and enrichment analysis. Among them, 19 significant DEGs, including 4 *PAL*, 1 *C4H*, 3 *4CL*, 1 *CHS*, 1 *DFR*, 1 *F3H*, 1 *FNSI*, 1 *FLS*, 2 *IFR*, and 4 *UGT*, were significantly up-regulated in S1 vs. S2 and S2 vs. S3 (Figure 7B), which further confirmed the results of metabolic profiling in chayote fruits during storage. Moreover, UDP-glycosyltransferases (*UGTs*) were involved in the synthesis of flavonoid glycosides in many plants (66, 67). In our study, a total of 47 *UGT*s were differentially expressed in S1, S2, and S3. Of these, four *UGT* genes were remarkably upregulated in S1 vs. S2 and S2 vs. S3, which was consistent with the results of metabolite profiling in chayote fruits during storage, indicating that these four *UGT* genes could catalyze the formation of flavonoid glycosides. Numerous studies have reported that *UGT* genes were involved in the biosynthesis of flavonoid glycosides (68–70), which was consistent with our results. Moreover, Yu et al. (69) reported that low temperature can induce the expression of *UGT g*ene in mulberry leaves, and result in the accumulation of flavonoid glycosides. Xie et al. (68) showed that two *UGT* genes, *MdUGT75B1* and *MdUGT71B1*, are involved in flavonol biosynthesis and may have important roles in regulating accumulation of these health-promoting bioactive compounds in apple.

### 4.4. Transcriptional factors involved in regulating the flavonoid synthesis

Previous studies had revealed that MYB, WRKY, bHLH, bZIP, and DOF TFs were involved in regulating the flavonoid synthesis (35–39). In present study, 45 MYB, 33 bHLH, 25 WRKY, 20 bZIP, and 22 Dof TFs with the differential expression patterns in chayote fruits at three differential storage stages (Supplementary Table S9). Among them, the expression patterns of 24 MYB, 14 bHLH, 10 WRKY, 11 bZIP, and 8 Dof TFs displayed a good linear relationship with structural genes in flavonoid synthesis pathway, which were increased or decreased in S1 vs. S2 and S2 vs. S3, suggesting that these TFs played important roles in regulating the flavonoid synthesis of chayote fruits. Of these 24 MYB, seven MYB TFs, including 4 positive regulatory TFs (FSG0019590, FSG0221650, FSG0057100, and FSG0036280) and 3 negative regulatory TFs (FSG0159340, FSG0079980, and FSG0179850), belong to R2R3-MYB subfamily, which have been reported to be involved in flavonoid biosynthesis. Moreover, the homologous genes of seven MYB TFs in *Arabidopsis* were MYB86, MYB73, MYB12, MYB78, MYB36, MYB78, MYB62, and MYB116, respectively. Previous studies revealed that seven R2R3-MYB TFs (MYB86, MYB73, MYB12, MYB78, MYB36, MYB78, MYB62, and MYB116) were involved in the positive/negative regulation of flavonoid biosynthesis (71–75). For example, Li et al. (76) reported that overexpression of SmMYB86 in eggplant reduced the accumulation of anthocyanins by decreasing the expression of *SmCHS*, *SmF3H*, and *SmANS*. Mehrtens et al. (77) revealed that MYB12, as a transcriptional regulator of *CHS* and *FLS*, acted as the flavonol-specific activator of flavonoid biosynthesis. With the development and improvement of gene knockout and transgenic technologies, overexpression and knockout of the selected candidate genes can change the flavonoid content by regulating the gene expression, which can improve the quality, enhance the tolerance to biotic and abiotic stress and extend shelf-life.

Correlation analysis between RNA-seq analysis and metabolite profiling indicated that the expression patterns of 18 DEGs (3 *PAL*, 1 *C4H*, 3 *4CL*, 1 *CHS*, 1 *DFR*, 1 *F3H*, 1 *FNSI*, 1 *FLS*, 2 *IFR* and 4 *UGT*) are closely correlated with the dynamic changes of 13 flavonoids, suggesting that these DEGs may be involved in regulating the flavonoid synthesis in chayote fruits during storage (Supplementary Table S11). Correlation analysis also revealed that 21 MYB and 12 bHLH TFs are closely correlated with eight flavonoid derivatives (*r* > 0.8; Figures 9B, C; Supplementary Table S12). In addition, correlation analysis between transcriptome and metabolite profiling also revealed that one R2R3-MYB TF (SeMYB1, FSG0057100) displayed a better correlation with five flavonoid glycosides, and the homologous gene of FSG0057100 in *Arabidopsis* was AtMYB12. Previous studies had revealed that MYB12 have the potential to activate *UGT* genes known to play a role in the formation of specific flavonol glycosides (39). This result indicated that FSG0057100 is the key regulators of the flavonoid synthesis in chayote fruits based on the expression pattern and correlation analysis.

In this study, the characterization and kinetic pattern of flavonoid constituents and differential flavonoid metabolites were identified in chayote fruits during storage. The DEGs involved in the flavonoid biosynthetic pathway were also detected using RNA-deq data. The expression pattern of some DEGs were further investigated using qRT-PCR. Moreover, correlation analysis between RNA-seq and metabolite revealed the structure genes and some TFs influencing flavonoid accumulation in chayote fruits during storage. Our results enable a comprehensive and systematic understanding of flavonoid compositions and dynamic changes as well as the corresponding molecular regulation mechanisms of flavonoid synthesis in chayote fruits.

## Data availability statement

The original contributions presented in the study are publicly available. This data can be found at: NCBI GEO, GSE214641.

## Author contributions

XW, YA, and WZ conceived and designed the experiments. YP and CW performed the experiment. XW, YP, WZ, and YJ analyzed the data. XW and YP wrote the manuscript. All authors read and approved the manuscript.

## Funding

This work was supported by the Science and Technology Project of Guizhou Province (Qiankehe Foundation-ZK [2022]), National Natural Science Foundation of China (32102306). This work was also supported by Talent Introduction Scientific Research Start-Up Project of Guizhou University (703/702735193301).

## Conflict of interest

The authors declare that the research was conducted in the absence of any commercial or financial relationships that could be construed as a potential conflict of interest.

## Publisher’s note

All claims expressed in this article are solely those of the authors and do not necessarily represent those of their affiliated organizations, or those of the publisher, the editors and the reviewers. Any product that may be evaluated in this article, or claim that may be made by its manufacturer, is not guaranteed or endorsed by the publisher.

## Footnotes

^1^ http://www.hmdb.ca/

^2^ https://metlin.scripps.edu/

^3^ https://cloud.majorbio.com
