# Supplementary material for: Metabolic profiling and transcriptome analysis provide insights into the accumulation of flavonoids in chayote fruit during storage
Source: Front Nutr. 2023 Feb 27;10:1029745. doi: 10.3389/fnut.2023.1029745 (PMC10019507; doi:10.3389/fnut.2023.1029745)
Supplement: Supplementary file 2 [file Table_1.docx]

**Supplementary Table 1 |** Primers used in this study

| Gene | Gene Locus | Forward primer | Reverse primer |
| --- | --- | --- | --- |
| SePAL 2 | FSG0199650.1 | GGGCAGACTCTCACTATTTC | CCTCCATGTATCGCTTTATTTC |
| SeC4H 1 | FSG0025420.1 | ACGTCGTCTTCGATATCTTC | GCTTGATTCTCGTCTCCTTA |
| Se4CL 2 | FSG0140440.1 | CTTCTTCTAACCCACTCATCTA | TCCTCGGTAAGTTCATTCTC |
| SeCHS 1 | FSG0098480.1 | CGGACTACTACTTTCGGATTAC | CAGAATCTCCTCCGTTAGATG |
| SeUGT 1 | FSG0049810.1 | ACTATGATCCGTACCTCAAAC | CAACAACATCGAAGAGCTTAC |
| SeF3H | FSG0060970.1 | GATGGACTTGGCCTCTAAAT | CGAGATCTGAGTTGGAACAT |
| SeFLS 1 | FSG0080030.1 | GATTTCAGGAACCCAGATCA | GAGCTCGAAGAACTCCTTAC |
| SeIFR2 | FSG0221220.1 | CCACTCGATGTTTATTAAGG | CAGTGGTGTATTTAACTTCC |
| SeUGT 4 | FSG0092410.1 | GGGAGATGAGATTGTGGATTA | CGAGACCATTACCAGAGAAG |
| SeDFR | FSG0149470.1 | CAGCTGTGAACGAAAGAATAG | GTATGCTCCATCAGGAAGAT |
| SeIFR 1 | FSG0001040.1 | CAATTCCTCTCCAAGATGAAAG | CTTCTTCTCCTTCGCCTAAT |
| SeFNSI1 | FSG0165280.1 | GCAGACCCACTCCAGTTTAG | CACCATCCTTAAGCCCATACTC |
| SeFNSI2 | FSG0165310.1 | GATCTGCCGCAACTTACCTATC | GGGATATGGTAGCCGTTGATTT |
| SeMYB1 | FSG0057100.1 | GCACTACTCTTCTTCTTCTTC | CAAGACGAAGGCTCAATAAA |
| SeMYB2 | FSG0023770.1 | GGAGAAGAAGAAAGTGGCTA | CTTGGGTTGCCCTAAATAATC |
| SeMYB3 | FSG0239060.1 | CCTCTGAATCTACCATCTCC | GCATTACCTTCTCTGCTTTC |
| SebHLH1 | FSG0242730.1 | CATAGCCTCAGCAAGTGATA | GGAGTTCATCCTCCCATAAC |
| SebHLH2 | FSG0125260.1 | CTCTCCATGCTCAGATCTAT | CTAGCAGTTCTTTCACATACTC |
| Actin7 | FSG0209090.1 | GAGGTTACATGTTCACTACTAC | GTCTCGAGTTCTTGTTCATAG |
