# Supplementary material for: Metabolic profiling and transcriptome analysis provide insights into the accumulation of flavonoids in chayote fruit during storage
Source: Front Nutr. 2023 Feb 27;10:1029745. doi: 10.3389/fnut.2023.1029745 (PMC10019507; doi:10.3389/fnut.2023.1029745)
Supplement: Supplementary file 3 [file Table_2.docx]

**Supplementary Table 2 |** The differential accumulation of flavonoid metabolites in S1 *vs.* S2

|  | Metab ID | Subtype | S1-1 | S1-1 | S1-1 | S2-1 | S2-2 | S2-3 |
| --- | --- | --- | --- | --- | --- | --- | --- | --- |
| Up | metab_10237 | Flavonoid glycosides | 2.3142 | 2.3084 | 2.3467 | 2.2785 | 2.4648 | 1.9272 |
|  | metab_10380 | Flavonoid glycosides | 1.6995 | 1.5072 | 1.3391 | 0.4496 | 0.6879 | 0.5803 |
|  | metab_10472 | Flavonoid glycosides | 2.2852 | 2.1976 | 2.0650 | 1.2281 | 1.2943 | 1.7700 |
|  | metab_10513 | Flavonoid glycosides | 3.1359 | 3.0141 | 2.9378 | 2.4773 | 2.3871 | 2.7729 |
|  | metab_10647 | Flavonoid glycosides | 1.6281 | 0.8544 | 0.9544 | 1.6224 | 1.1287 | 1.4606 |
|  | metab_14871 | Flavonoid glycosides | 3.0654 | 2.8983 | 2.8741 | 2.2778 | 2.3106 | 2.5149 |
|  | metab_15089 | Isoflavonoid O-glycosides | 1.3854 | 1.2515 | 1.4789 | 0.1442 | 0.1301 | 0.3037 |
|  | metab_15145 | Flavonoid glycosides | 2.6615 | 2.5722 | 2.4924 | 2.1566 | 2.1446 | 2.3678 |
|  | metab_15176 | Flavonoid glycosides | 3.0662 | 2.9986 | 2.9360 | 2.5549 | 2.5958 | 2.7504 |
|  | metab_15297 | Flavonoid glycosides | 1.6617 | 1.5464 | 1.5275 | 0.8812 | 0.5235 | 0.9559 |
|  | metab_15311 | Flavonoid glycosides | 1.5750 | 1.2910 | 1.4211 | 1.2909 | 1.2564 | 1.3671 |
|  | metab_15346 | Flavonoid glycosides | 3.8530 | 3.7190 | 3.7156 | 3.2080 | 3.0689 | 3.5566 |
|  | metab_15368 | Flavonoid glycosides | 2.2433 | 2.0005 | 1.7726 | 1.0318 | 0.9966 | 1.0107 |
|  | metab_2581 | Prenylated neoflavonoids | 1.8773 | 1.9435 | 2.1118 | 1.4227 | 1.4990 | 1.2669 |
|  | metab_648 | Flavonoid glycosides | 3.3069 | 3.1163 | 3.0264 | 1.2358 | 0.9939 | 2.1534 |
|  | metab_6688 | Flavonoid glycosides | 1.3845 | 1.3210 | 1.4285 | 0.8521 | 0.7813 | 0.8069 |
|  | metab_6862 | Flavonoid glycosides | 3.7083 | 3.6524 | 3.7182 | 3.5183 | 3.5443 | 3.7495 |
|  | metab_7028 | Flavonoid glycosides | 1.7772 | 1.5927 | 1.6720 | 0.6608 | 1.2027 | 0.9125 |
|  | metab_7064 | Flavonoid glycosides | 3.3666 | 3.2822 | 3.3034 | 2.7288 | 2.6086 | 3.0989 |
|  | metab_7120 | Flavonoid glycosides | 2.5014 | 2.4220 | 2.3902 | 1.7958 | 1.4779 | 1.8311 |
|  | metab_7267 | Flavonoid glycosides | 2.0980 | 1.9933 | 1.7133 | 0.0990 | 0.0936 | 0.1075 |
|  | metab_7269 | Pyranoisoflavonoids | 2.4753 | 2.6031 | 2.6215 | 2.2286 | 1.9449 | 2.2334 |
|  | metab_7512 | Furanoisoflavonoids | 2.4632 | 2.4073 | 2.3804 | 2.2600 | 2.2442 | 2.2585 |
|  | metab_8428 | Isoflavonoid O-glycosides | 2.2450 | 2.1066 | 2.0721 | 1.7211 | 1.7296 | 1.5426 |
| Down | metab_6431 | O-methylated flavonoids | 1.1947 | 1.1492 | 1.2002 | 1.6350 | 1.6935 | 1.3705 |
|  | metab_8444 | Flavonoid glycosides | 2.2196 | 2.1034 | 2.0922 | 2.0476 | 2.0508 | 2.2646 |
|  | metab_2739 | O-methylated isoflavonoids | 0.8941 | 1.2183 | 1.3842 | 1.7619 | 1.9537 | 1.8576 |
|  | metab_10685 | Flavones | 1.5574 | 1.4198 | 0.9948 | 2.4377 | 1.6893 | 3.1432 |
|  | metab_14725 | Flavonoid glycosides | 0.3071 | 0.2639 | 0.5011 | 2.3764 | 2.3605 | 2.6433 |
|  | metab_6565 | Flavonoid glycosides | 0.6316 | 0.6156 | 0.6074 | 2.4318 | 1.3355 | 3.2594 |
|  | metab_14662 | Flavonoid glycosides | 0.6206 | 0.5870 | 0.6016 | 1.7561 | 1.7792 | 1.6063 |
|  | metab_14970 | Flavonoid glycosides | 1.2165 | 0.4464 | 0.9176 | 2.2755 | 1.6855 | 2.4627 |
|  | metab_1205 | Isoflavonoid O-glycosides | 1.4806 | 1.4668 | 1.4888 | 1.9080 | 1.9857 | 1.8764 |
|  | metab_640 | Flavonoid glycosides | 4.7264 | 4.7262 | 4.7516 | 4.9633 | 4.9416 | 5.1066 |
|  | metab_1269 | Flavonoid glycosides | 1.4782 | 1.2355 | 1.3730 | 2.0197 | 2.0577 | 1.8624 |
|  | metab_6733 | Flavonoid glycosides | 0.9864 | 0.5416 | 0.7978 | 1.5509 | 1.1643 | 2.0532 |
|  | metab_14666 | Flavonoid glycosides | 2.7407 | 2.6005 | 2.6378 | 2.9943 | 3.0033 | 2.8730 |
