# Supplementary material for: Metabolic profiling and transcriptome analysis provide insights into the accumulation of flavonoids in chayote fruit during storage
Source: Front Nutr. 2023 Feb 27;10:1029745. doi: 10.3389/fnut.2023.1029745 (PMC10019507; doi:10.3389/fnut.2023.1029745)
Supplement: Supplementary file 4 [file Table_3.docx]

**Supplementary Table 3** | The differential accumulation of flavonoid metabolites in S1 *vs.* S3

|  | Metab ID | subtype | S1-1 | S1-1 | S1-1 | S3-1 | S3-2 | S3-3 |
| --- | --- | --- | --- | --- | --- | --- | --- | --- |
| Up | metab_15368 | Flavonoid glycosides | 2.2433 | 2.0005 | 1.7726 | 2.1151 | 2.2146 | 3.3237 |
|  | metab_2750 | Flavonoid glycosides | 1.9750 | 1.9402 | 1.9173 | 1.5193 | 1.6555 | 1.6717 |
|  | metab_10472 | Flavonoid glycosides | 2.2852 | 2.1976 | 2.0650 | 1.7709 | 1.8360 | 2.0511 |
|  | metab_14871 | Flavonoid glycosides | 3.0654 | 2.8983 | 2.8741 | 2.5465 | 2.4669 | 2.6723 |
|  | metab_6688 | Flavonoid glycosides | 1.3845 | 1.3210 | 1.4285 | 0.7527 | 0.8054 | 0.9752 |
|  | metab_7173 | Flavonoid glycosides | 2.1355 | 2.1361 | 2.2240 | 0.8504 | 0.8409 | 0.7194 |
|  | metab_8444 | Flavonoid glycosides | 2.2196 | 2.1034 | 2.0922 | 2.1328 | 2.0687 | 2.1148 |
|  | metab_2581 | Prenylated neoflavonoids | 1.8773 | 1.9435 | 2.1118 | 1.2922 | 0.7607 | 1.2728 |
|  | metab_10464 | Flavonoid glycosides | 2.2501 | 2.2168 | 2.1463 | 1.9321 | 1.4591 | 1.3261 |
|  | metab_15591 | Flavonoid glycosides | 1.2252 | 1.3305 | 1.6099 | 0.3675 | 0.5912 | 0.2155 |
|  | metab_8428 | Isoflavonoid O-glycosides | 2.2450 | 2.1066 | 2.0721 | 1.4332 | 1.1802 | 1.4275 |
|  | metab_648 | Flavonoid glycosides | 3.3069 | 3.1163 | 3.0264 | 0.7966 | 0.5616 | 1.1283 |
|  | metab_7064 | Flavonoid glycosides | 3.3666 | 3.2822 | 3.3034 | 2.7675 | 2.8825 | 2.8531 |
|  | metab_7120 | Flavonoid glycosides | 2.5014 | 2.4220 | 2.3902 | 0.8550 | 0.9060 | 0.5690 |
|  | metab_15297 | Flavonoid glycosides | 1.6617 | 1.5464 | 1.5275 | 0.5545 | 0.4402 | 0.4736 |
|  | metab_15535 | Flavonoid glycosides | 3.5618 | 3.5464 | 3.5065 | 2.8372 | 2.7448 | 2.9804 |
|  | metab_15484 | Flavonoid glycosides | 3.5031 | 3.3712 | 3.3601 | 2.3912 | 2.6602 | 2.2241 |
|  | metab_6862 | Flavonoid glycosides | 3.7083 | 3.6524 | 3.7182 | 3.5451 | 3.5552 | 3.7531 |
|  | metab_10513 | Flavonoid glycosides | 3.1359 | 3.0141 | 2.9378 | 2.6597 | 2.5320 | 2.7008 |
|  | metab_10647 | Flavonoid glycosides | 1.6281 | 0.8544 | 0.9544 | 0.2899 | 0.8017 | 0.7773 |
|  | metab_10826 | Isoflavans | 1.7233 | 1.7629 | 1.6311 | 0.3715 | 0.9794 | 0.4445 |
|  | metab_5745 | O-methylated flavonoids | 1.7853 | 1.8218 | 1.6226 | 0.4328 | 0.5255 | 0.4199 |
|  | metab_7267 | Flavonoid glycosides | 2.0980 | 1.9933 | 1.7133 | 0.1044 | 0.0928 | 0.7836 |
|  | metab_7269 | Pyranoisoflavonoids | 2.4753 | 2.6031 | 2.6215 | 0.9367 | 0.9455 | 0.9159 |
|  | metab_15346 | Flavonoid glycosides | 3.8530 | 3.7190 | 3.7156 | 3.1014 | 3.0705 | 2.9760 |
| Down | metab_14140 | Flavonoid glycosides | 1.7535 | 1.6414 | 1.9495 | 2.2583 | 2.1365 | 2.0860 |
|  | metab_1269 | Flavonoid glycosides | 1.4782 | 1.2355 | 1.3730 | 1.9909 | 2.0604 | 1.9704 |
|  | metab_10538 | Flavonoid glycosides | 1.8525 | 1.6766 | 1.6689 | 2.6661 | 2.4735 | 2.3724 |
|  | metab_1205 | Isoflavonoid O-glycosides | 1.4806 | 1.4668 | 1.4888 | 1.9404 | 1.9391 | 1.8451 |
|  | metab_14662 | Flavonoid glycosides | 0.6206 | 0.5870 | 0.6016 | 1.7621 | 1.6672 | 1.5919 |
|  | metab_7512 | Furanoisoflavonoids | 2.4632 | 2.4073 | 2.3804 | 2.6695 | 2.5282 | 2.6494 |
|  | metab_14666 | Flavonoid glycosides | 2.7407 | 2.6005 | 2.6378 | 2.9985 | 2.9050 | 2.8454 |
|  | metab_6733 | Flavonoid glycosides | 0.9864 | 0.5416 | 0.7978 | 1.8865 | 1.9215 | 2.2483 |
|  | metab_14970 | Flavonoid glycosides | 1.2165 | 0.4464 | 0.9176 | 2.3867 | 2.3327 | 2.5496 |
|  | metab_7028 | Flavonoid glycosides | 1.7772 | 1.5927 | 1.6720 | 2.1882 | 1.6472 | 2.3114 |
|  | metab_640 | Flavonoid glycosides | 4.7264 | 4.7262 | 4.7516 | 4.9693 | 5.0214 | 5.0113 |
|  | metab_6944 | Flavonoid glycosides | 2.2904 | 2.3315 | 2.1370 | 2.5006 | 2.7905 | 2.8176 |
|  | metab_6431 | O-methylated flavonoids | 1.1947 | 1.1492 | 1.2002 | 1.5346 | 1.5560 | 1.6449 |
|  | metab_2739 | O-methylated isoflavonoids | 0.8941 | 1.2183 | 1.3842 | 1.5358 | 1.3905 | 1.4931 |
|  | metab_15311 | Flavonoid glycosides | 1.5750 | 1.2910 | 1.4211 | 2.0743 | 1.9390 | 2.1863 |
