# Supplementary material for: Metabolic profiling and transcriptome analysis provide insights into the accumulation of flavonoids in chayote fruit during storage
Source: Front Nutr. 2023 Feb 27;10:1029745. doi: 10.3389/fnut.2023.1029745 (PMC10019507; doi:10.3389/fnut.2023.1029745)
Supplement: Supplementary file 5 [file Table_4.docx]

**Supplementary Table 4 |** The differential accumulation of flavonoid metabolites in S2 *vs.* S3

|  | Metab ID | Subtype | S2-1 | S2-2 | S2-3 | S3-1 | S3-2 | S3-3 |
| --- | --- | --- | --- | --- | --- | --- | --- | --- |
| Down | metab_10237 | Flavonoid glycosides | 2.2785 | 2.4648 | 1.9272 | 2.3669 | 2.3179 | 2.2767 |
|  | metab_7512 | Furanoisoflavonoids | 2.2600 | 2.2442 | 2.2585 | 2.6695 | 2.5282 | 2.6494 |
|  | metab_15368 | Flavonoid glycosides | 1.0318 | 0.9966 | 1.0107 | 2.1151 | 2.2146 | 3.3237 |
|  | metab_15176 | Flavonoid glycosides | 2.5549 | 2.5958 | 2.7504 | 3.0878 | 2.9951 | 3.1553 |
|  | metab_15311 | Flavonoid glycosides | 1.2909 | 1.2564 | 1.3671 | 2.0743 | 1.9390 | 2.1863 |
|  | metab_10538 | Flavonoid glycosides | 1.7986 | 1.6173 | 1.9873 | 2.6661 | 2.4735 | 2.3724 |
|  | metab_7028 | Flavonoid glycosides | 0.6608 | 1.2027 | 0.9125 | 2.1882 | 1.6472 | 2.3114 |
|  | metab_15145 | Flavonoid glycosides | 2.1566 | 2.1446 | 2.3678 | 2.6790 | 2.5293 | 2.6562 |
| Up | metab_7269 | Pyranoisoflavonoids | 2.2286 | 1.9449 | 2.2334 | 0.9367 | 0.9455 | 0.9159 |
|  | metab_15591 | Flavonoid glycosides | 1.2676 | 0.8726 | 1.1831 | 0.3675 | 0.5912 | 0.2155 |
|  | metab_10464 | Flavonoid glycosides | 1.8953 | 2.0054 | 1.9566 | 1.9321 | 1.4591 | 1.3261 |
|  | metab_5745 | O-methylated flavonoids | 1.3541 | 1.0356 | 1.6080 | 0.4328 | 0.5255 | 0.4199 |
|  | metab_15297 | Flavonoid glycosides | 0.8812 | 0.5235 | 0.9559 | 0.5545 | 0.4402 | 0.4736 |
|  | metab_648 | Flavonoid glycosides | 1.2358 | 0.9939 | 2.1534 | 0.7966 | 0.5616 | 1.1283 |
|  | metab_15346 | Flavonoid glycosides | 3.2080 | 3.0689 | 3.5566 | 3.1014 | 3.0705 | 2.9760 |
|  | metab_10826 | Isoflavans | 1.3474 | 0.8025 | 1.4883 | 0.3715 | 0.9794 | 0.4445 |
|  | metab_10647 | Flavonoid glycosides | 1.6224 | 1.1287 | 1.4606 | 0.2899 | 0.8017 | 0.7773 |
|  | metab_15484 | Flavonoid glycosides | 3.2075 | 3.0097 | 3.4073 | 2.3912 | 2.6602 | 2.2241 |
|  | metab_6862 | Flavonoid glycosides | 3.5183 | 3.5443 | 3.7495 | 3.5451 | 3.5552 | 3.7531 |
|  | metab_7064 | Flavonoid glycosides | 2.7288 | 2.6086 | 3.0989 | 2.7675 | 2.8825 | 2.8531 |
|  | metab_15535 | Flavonoid glycosides | 3.1184 | 2.9548 | 3.5039 | 2.8372 | 2.7448 | 2.9804 |
|  | metab_640 | Flavonoid glycosides | 4.9633 | 4.9416 | 5.1066 | 4.9693 | 5.0214 | 5.0113 |
|  | metab_8444 | Flavonoid glycosides | 2.0476 | 2.0508 | 2.2646 | 2.1328 | 2.0687 | 2.1148 |
|  | metab_7120 | Flavonoid glycosides | 1.7958 | 1.4779 | 1.8311 | 0.8550 | 0.9060 | 0.5690 |
|  | metab_7173 | Flavonoid glycosides | 1.9558 | 1.7279 | 2.2205 | 0.8504 | 0.8409 | 0.7194 |
|  | metab_14725 | Flavonoid glycosides | 2.3764 | 2.3605 | 2.6433 | 0.6788 | 0.6887 | 0.5367 |
|  | metab_8428 | Isoflavonoid O-glycosides | 1.7211 | 1.7296 | 1.5426 | 1.4332 | 1.1802 | 1.4275 |
|  | metab_10685 | Flavones | 2.4377 | 1.6893 | 3.1432 | 1.1789 | 1.3481 | 1.6005 |
