# Supplementary material for: Metabolic profiling and transcriptome analysis provide insights into the accumulation of flavonoids in chayote fruit during storage
Source: Front Nutr. 2023 Feb 27;10:1029745. doi: 10.3389/fnut.2023.1029745 (PMC10019507; doi:10.3389/fnut.2023.1029745)
Supplement: Supplementary file 6 [file Table_5.docx]

**Supplementary Table 5** The differential accumulation of flavonoid metabolites in S1 *vs.* S2, S1 *vs.* S3 and S2 *vs.* S3

| Metab ID | Subtype | S1-1 | S1-1 | S1-1 | S2-1 | S2-2 | S2-3 | S3-1 | S3-2 | S3-3 |
| --- | --- | --- | --- | --- | --- | --- | --- | --- | --- | --- |
| metab_7512 | Furanoisoflavonoids | 2.4632 | 2.4073 | 2.3804 | 2.2600 | 2.2442 | 2.2585 | 2.6695 | 2.5282 | 2.6494 |
| metab_15368 | Flavonoid glycosides | 2.2433 | 2.0005 | 1.7726 | 1.0318 | 0.9966 | 1.0107 | 2.1151 | 2.2146 | 3.3237 |
| metab_15311 | Flavonoid glycosides | 1.5750 | 1.2910 | 1.4211 | 1.2909 | 1.2564 | 1.3671 | 2.0743 | 1.9390 | 2.1863 |
| metab_7028 | Flavonoid glycosides | 1.7772 | 1.5927 | 1.6720 | 0.6608 | 1.2027 | 0.9125 | 2.1882 | 1.6472 | 2.3114 |
| metab_7269 | Pyranoisoflavonoids | 2.4753 | 2.6031 | 2.6215 | 2.2286 | 1.9449 | 2.2334 | 0.9367 | 0.9455 | 0.9159 |
| metab_15297 | Flavonoid glycosides | 1.6617 | 1.5464 | 1.5275 | 0.8812 | 0.5235 | 0.9559 | 0.5545 | 0.4402 | 0.4736 |
| metab_648 | Flavonoid glycosides | 3.3069 | 3.1163 | 3.0264 | 1.2358 | 0.9939 | 2.1534 | 0.7966 | 0.5616 | 1.1283 |
| metab_15346 | Flavonoid glycosides | 3.8530 | 3.7190 | 3.7156 | 3.2080 | 3.0689 | 3.5566 | 3.1014 | 3.0705 | 2.9760 |
| metab_10647 | Flavonoid glycosides | 1.6281 | 0.8544 | 0.9544 | 1.6224 | 1.1287 | 1.4606 | 0.2899 | 0.8017 | 0.7773 |
| metab_6862 | Flavonoid glycosides | 3.7083 | 3.6524 | 3.7182 | 3.5183 | 3.5443 | 3.7495 | 3.5451 | 3.5552 | 3.7531 |
| metab_7064 | Flavonoid glycosides | 3.3666 | 3.2822 | 3.3034 | 2.7288 | 2.6086 | 3.0989 | 2.7675 | 2.8825 | 2.8531 |
| metab_640 | Flavonoid glycosides | 4.7264 | 4.7262 | 4.7516 | 4.9633 | 4.9416 | 5.1066 | 4.9693 | 5.0214 | 5.0113 |
| metab_8444 | Flavonoid glycosides | 2.2196 | 2.1034 | 2.0922 | 2.0476 | 2.0508 | 2.2646 | 2.1328 | 2.0687 | 2.1148 |
| metab_7120 | Flavonoid glycosides | 2.5014 | 2.4220 | 2.3902 | 1.7958 | 1.4779 | 1.8311 | 0.8550 | 0.9060 | 0.5690 |
| metab_8428 | Isoflavonoid O-glycosides | 2.2450 | 2.1066 | 2.0721 | 1.7211 | 1.7296 | 1.5426 | 1.4332 | 1.1802 | 1.4275 |
