# Supplementary material for: Metabolic profiling and transcriptome analysis provide insights into the accumulation of flavonoids in chayote fruit during storage
Source: Front Nutr. 2023 Feb 27;10:1029745. doi: 10.3389/fnut.2023.1029745 (PMC10019507; doi:10.3389/fnut.2023.1029745)
Supplement: Supplementary file 8 [file Table_7.docx]

**Supplementary Table 7 |** The differential structure genes associated with flavonoid biosynthesis in chayote fruits during storage

| Gene_id | S1-1 | S1-2 | S1-3 | S2-1 | S2-2 | S2-3 | S3-1 | S3-2 | S3-3 |
| --- | --- | --- | --- | --- | --- | --- | --- | --- | --- |
| SePAL1 | 2.12 | 4.71 | 1.53 | 5.48 | 17.87 | 41.49 | 1.24 | 0.12 | 0.24 |
| SePAL2 | 1.38 | 1.2 | 1.09 | 2.51 | 2.06 | 3.1 | 0.1 | 0.4 | 0 |
| SePAL3 | 1.24 | 1.88 | 0.9 | 0.84 | 0.69 | 4.25 | 1.36 | 0.82 | 0.32 |
| SePAL4 | 17.31 | 19.96 | 16.6 | 30.49 | 58.84 | 47.12 | 18.41 | 3.3 | 5.85 |
| SePAL5 | 0.57 | 0.29 | 0.26 | 4.47 | 9.13 | 10.3 | 0.31 | 0.27 | 0.23 |
| SePAL6 | 3.45 | 16.97 | 6.7 | 5.25 | 10.94 | 10.33 | 6.35 | 0.36 | 1.37 |
| SePAL7 | 0.41 | 0 | 0.28 | 0.6 | 0 | 0.96 | 0.15 | 0 | 0.16 |
| SePAL8 | 7.89 | 9.54 | 9.69 | 1.32 | 1.88 | 1.66 | 7.76 | 2.29 | 2.38 |
| SePAL9 | 1.72 | 5.55 | 1.56 | 6.62 | 26.57 | 47.8 | 2.6 | 0.19 | 0.16 |
| SePAL10 | 2.14 | 4.12 | 2.08 | 0.61 | 0.79 | 4.11 | 1.01 | 0.52 | 0.77 |
| SePAL11 | 1.85 | 2.34 | 2.36 | 3.52 | 4.41 | 5.17 | 2.21 | 0.33 | 0.29 |
| SePAL12 | 17.31 | 19.96 | 16.6 | 30.49 | 58.84 | 47.12 | 18.41 | 3.3 | 5.85 |
| SePAL13 | 10.4 | 7.87 | 8.01 | 17.76 | 25.54 | 14.59 | 9.6 | 4.37 | 3.44 |
| SePAL14 | 2.14 | 0 | 0.61 | 1.36 | 0.37 | 0 | 0 | 0.79 | 0 |
| SePAL15 | 18.06 | 17.93 | 18.48 | 22.91 | 22.04 | 11.5 | 16.85 | 10.86 | 5.38 |
| SePAL16 | 8.19 | 7.54 | 6.8 | 1.01 | 1.07 | 0.96 | 4.45 | 1.41 | 1.77 |
| SeC4H1 | 0.67 | 1.01 | 0.99 | 0 | 0 | 0 | 0 | 0 | 0 |
| SeC4H2 | 37.47 | 31.64 | 34.47 | 106.81 | 220.62 | 287.4 | 81.16 | 58.13 | 30.19 |
| Se4CL1 | 21.25 | 22.38 | 16.36 | 132.71 | 83.68 | 90 | 31.99 | 10.52 | 8.2 |
| Se4CL2 | 11.84 | 11.35 | 13.38 | 22.73 | 29.11 | 46.03 | 13.68 | 9.21 | 11.06 |
| Se4CL3 | 2.43 | 3.64 | 4.16 | 26.33 | 20.12 | 12.78 | 4.29 | 6.08 | 3.8 |
| Se4CL4 | 24.27 | 25.75 | 23.07 | 30.22 | 35.09 | 47.35 | 83.3 | 45.01 | 42.2 |
| Se4CL5 | 68.66 | 61.44 | 68.68 | 25.39 | 46.1 | 81.52 | 17.99 | 20.66 | 19.02 |
| Se4CL6 | 22.74 | 18.64 | 22.38 | 0.42 | 1.75 | 2.3 | 4.79 | 8.06 | 9.49 |
| Se4CL7 | 0 | 0 | 0.16 | 0 | 0 | 0.31 | 0 | 0.41 | 0.53 |
| Se4CL8 | 0 | 0 | 0 | 0 | 0 | 0 | 0.97 | 1.15 | 0 |
| Se4CL9 | 1.94 | 0.32 | 0.61 | 0.34 | 0.38 | 2.24 | 0 | 0 | 0 |
| Se4CL10 | 101.58 | 94.7 | 89.32 | 128.57 | 138.2 | 123.52 | 117.61 | 142.18 | 110.15 |
| Se4CL11 | 6.69 | 7.26 | 6.2 | 14.11 | 29.86 | 60.37 | 15.02 | 4.95 | 5.08 |
| Se4CL12 | 2.03 | 2.42 | 3.51 | 0.49 | 0.98 | 1.07 | 1.14 | 2.65 | 3.36 |
| Se4CL13 | 9.76 | 9.63 | 9.37 | 11.17 | 9 | 10.91 | 7.01 | 4.05 | 4.51 |
| Se4CL14 | 0.56 | 0.84 | 0.26 | 0.12 | 0.41 | 6.4 | 0.45 | 0.36 | 0.1 |
| Se4CL15 | 14.4 | 13.77 | 10.68 | 8.29 | 16.23 | 24.57 | 11.46 | 12.03 | 15.51 |
| Se4CL16 | 1.83 | 2.78 | 2.29 | 3.63 | 4.2 | 1.79 | 6.99 | 9.19 | 4.36 |
| SeCHS1 | 2.89 | 2.57 | 2.86 | 11.07 | 11.91 | 5.95 | 2.74 | 4.21 | 3.22 |
| SeCHS2 | 22.5 | 36.09 | 43.76 | 76.83 | 8.45 | 2.05 | 168 | 127.68 | 90.07 |
| SeDFR | 26.5 | 26.31 | 20.9 | 73.98 | 49.69 | 70.72 | 104.07 | 18.31 | 35.84 |
| SeF3H | 49.94 | 46.95 | 48.83 | 73.21 | 69.49 | 41.39 | 16.4 | 4.5 | 9.23 |
| SeFNSI1 | 4.92 | 9.35 | 5.15 | 1.64 | 1.73 | 3.56 | 0 | 0 | 0.03 |
| SeFNSI2 | 5.58 | 6.24 | 4.25 | 2.92 | 3.14 | 4.03 | 3.95 | 11.49 | 22.59 |
| SeFLS1 | 168.89 | 188.66 | 182.84 | 20.87 | 17.71 | 15.97 | 315 | 413.38 | 323.66 |
| SeFLS2 | 13.69 | 12.71 | 11.49 | 9.23 | 7.44 | 7.9 | 32.73 | 31.95 | 16.95 |
| SeFLS3 | 0.53 | 0.23 | 0.2 | 0.04 | 0.12 | 0.91 | 90.08 | 42.61 | 45.62 |
| SeIFR1 | 50.94 | 49.31 | 47.38 | 143.86 | 183.81 | 194.93 | 105.67 | 49.01 | 47.96 |
| SeIFR2 | 13.82 | 5.51 | 14.81 | 0.07 | 0 | 0.12 | 4.5 | 3.64 | 3.54 |
| SeIFR3 | 23.61 | 9.59 | 23.23 | 0.05 | 0.12 | 0.25 | 4.2 | 5.76 | 7.05 |
| SeIFR4 | 32.23 | 25.25 | 22.78 | 8.1 | 7.45 | 15.52 | 24.43 | 13.91 | 17.22 |
| SeIFR5 | 13.28 | 14.79 | 12.26 | 15 | 16.77 | 19.76 | 23.18 | 13.09 | 13 |
| SeUGT1 | 44.09 | 42.18 | 45.54 | 2.83 | 2.1 | 3.28 | 55.61 | 103.45 | 97.48 |
| SeUGT2 | 15.67 | 13.78 | 10.16 | 11.99 | 12.35 | 12.06 | 13.26 | 17.69 | 15.11 |
| SeUGT3 | 29.27 | 30.18 | 29.92 | 22.27 | 26.39 | 32.93 | 36.38 | 29.76 | 34.63 |
| SeUGT4 | 89.29 | 76.66 | 93.27 | 55.38 | 30.42 | 35.56 | 179.35 | 207.88 | 192.86 |
| SeUGT5 | 575.26 | 528.09 | 518.9 | 25.25 | 22.22 | 30.34 | 179.62 | 243.2 | 265.15 |
| SeUGT6 | 16.22 | 22.23 | 28.47 | 723.11 | 339.85 | 308.03 | 181.07 | 100.44 | 70.41 |
| SeUGT7 | 31.27 | 25.89 | 27.45 | 53.6 | 62.83 | 59.14 | 112.09 | 102.71 | 66.92 |
| SeUGT8 | 43.07 | 30.03 | 33.37 | 0 | 0 | 0.02 | 102.77 | 89.7 | 111.79 |
| SeUGT9 | 23.44 | 27.2 | 21.23 | 12.73 | 3.78 | 5.55 | 1.48 | 4.1 | 3.8 |
| SeUGT10 | 1.16 | 1.52 | 2.22 | 28.13 | 9.7 | 10 | 9.93 | 28.01 | 15.51 |
| SeUGT11 | 36.36 | 35.01 | 19.29 | 0.21 | 0.56 | 0.74 | 13.7 | 3.1 | 1.36 |
| SeUGT12 | 25.45 | 26.26 | 25.1 | 42.13 | 35.11 | 32.79 | 68.7 | 55.55 | 37.87 |
| SeUGT13 | 10.61 | 10.54 | 10.17 | 49.62 | 67.28 | 74.43 | 47.28 | 23.4 | 23.09 |
| SeUGT14 | 25.7 | 21.78 | 30.76 | 0 | 0 | 0 | 4.32 | 10.44 | 9.98 |
| SeUGT15 | 20.72 | 23.76 | 22.94 | 30.4 | 42.54 | 41.31 | 84.13 | 40.22 | 33.17 |
| SeUGT16 | 138.09 | 129.01 | 153.2 | 454.51 | 457.67 | 508.54 | 90.38 | 89.39 | 120.62 |
| SeUGT17 | 1.39 | 1.16 | 1.27 | 82.81 | 81.09 | 102.3 | 8.32 | 5.17 | 10.49 |
| SeUGT18 | 119.8 | 112.86 | 100.97 | 6 | 3.7 | 1.64 | 57.55 | 117.96 | 151.03 |
| SeUGT19 | 241.85 | 225.11 | 225.72 | 53.42 | 43.74 | 45.31 | 170.47 | 183.26 | 174.03 |
| SeUGT20 | 1.42 | 2.12 | 1.69 | 28.25 | 50.85 | 61.76 | 4.32 | 3.83 | 2.32 |
| SeUGT21 | 72.3 | 61.82 | 74.47 | 89.72 | 122.84 | 143.47 | 37.51 | 48.55 | 59.91 |
| SeUGT22 | 64.41 | 65.46 | 62.26 | 9.51 | 4.82 | 4.79 | 60.13 | 60.8 | 60.57 |
| SeUGT23 | 270.7 | 256.24 | 271.54 | 97.65 | 93.49 | 85.6 | 216.9 | 336.9 | 319.73 |
| SeUGT24 | 42.16 | 54.67 | 59.88 | 17.5 | 14.45 | 6.23 | 50.46 | 96.52 | 66.56 |
| SeUGT25 | 7 | 7.63 | 9.75 | 30.78 | 28.5 | 28.66 | 12.41 | 5.37 | 6.11 |
| SeUGT26 | 12.52 | 15.71 | 12.76 | 8.47 | 29.76 | 54.55 | 3.56 | 4.93 | 1.83 |
| SeUGT27 | 48.01 | 45.71 | 49.23 | 13.88 | 13.72 | 20.63 | 44.61 | 57.32 | 64.78 |
| SeUGT28 | 2.83 | 3.63 | 1.75 | 14.94 | 28.63 | 38.87 | 12.18 | 2.11 | 1.63 |
| SeUGT29 | 27.74 | 26.28 | 29.29 | 14.27 | 9.29 | 10.2 | 41.71 | 43.03 | 42.68 |
| SeUGT30 | 2.94 | 3.03 | 2.12 | 7.49 | 15.63 | 31.32 | 3.1 | 2.86 | 3.06 |
| SeUGT31 | 3.78 | 3.42 | 3.47 | 14.57 | 9.71 | 17.79 | 2.86 | 2.29 | 3.34 |
| SeUGT32 | 54.85 | 53.38 | 55.42 | 8.73 | 10.08 | 8.11 | 24.02 | 44.25 | 44.19 |
| SeUGT33 | 18.4 | 19.52 | 20.98 | 2.21 | 0.53 | 1.39 | 9.08 | 20.27 | 16.05 |
| SeUGT34 | 17.23 | 15.43 | 18.99 | 22.89 | 20.29 | 18.41 | 10.1 | 10.06 | 10.83 |
| SeUGT35 | 61.04 | 61.52 | 54.48 | 12.69 | 11.44 | 12.4 | 31.28 | 34.99 | 36.92 |
| SeUGT36 | 301 | 259.57 | 262.86 | 64.66 | 62.3 | 65.13 | 112.33 | 146.82 | 165.57 |
| SeUGT37 | 132.06 | 123.45 | 131.63 | 219.04 | 213.79 | 228.01 | 244.6 | 175.92 | 171.21 |
| SeUGT38 | 26.95 | 27.3 | 27.85 | 66.63 | 57.45 | 54 | 40.03 | 38.71 | 37.85 |
| SeUGT39 | 75.62 | 67.75 | 68.98 | 23.42 | 24.57 | 31.9 | 40.09 | 47.96 | 62.5 |
| SeUGT40 | 25.3 | 23.47 | 23.87 | 4.72 | 5.01 | 4.08 | 13.67 | 13.5 | 13.59 |
| SeUGT41 | 17 | 13.44 | 9.89 | 0.04 | 0.16 | 0.69 | 6.25 | 3.75 | 2.48 |
| SeUGT42 | 27.47 | 31.28 | 28.34 | 52.52 | 45.38 | 43.03 | 33.89 | 43.74 | 39.78 |
| SeUGT43 | 10.28 | 10.08 | 10.47 | 18.81 | 21.16 | 22.47 | 32.54 | 24.51 | 21.93 |
| SeUGT44 | 44.84 | 42.92 | 45.64 | 15.88 | 17.04 | 17.1 | 25.08 | 26.37 | 27.58 |
| SeUGT45 | 9.89 | 8 | 4.09 | 0 | 0.04 | 0 | 1.99 | 0.47 | 0.17 |
| SeUGT46 | 13.51 | 14.62 | 13.81 | 33.33 | 24.52 | 19.6 | 27.07 | 19.91 | 22.08 |
| SeUGT47 | 32.08 | 39.9 | 49.89 | 14.43 | 16.05 | 13.4 | 18.71 | 25.67 | 26.64 |
