# Supplementary material for: Metabolic profiling and transcriptome analysis provide insights into the accumulation of flavonoids in chayote fruit during storage
Source: Front Nutr. 2023 Feb 27;10:1029745. doi: 10.3389/fnut.2023.1029745 (PMC10019507; doi:10.3389/fnut.2023.1029745)
Supplement: Supplementary file 9 [file Table_8.docx]

**Supplementary Table 8 |** The structure genes of involved positive and negtive regulation associated with flavonoid biosynthesis in chayote fruits during storage

| Differential expression | ID | Unigene | Gene | S1 | S2 | S3 |
| --- | --- | --- | --- | --- | --- | --- |
| Positive regulation | SePAL1 | TRINITY_DN1395_c0_g2 | FSG0087710 | 21.61 | 2.79 | 0.53 |
|  | SePAL2 | TRINITY_DN13591_c0_g1 | FSG0272410 | 2.56 | 1.22 | 0.17 |
|  | SePAL3 | TRINITY_DN5659_c0_g2 | FSG0199650 | 1.93 | 1.34 | 0.83 |
|  | SePAL4 | TRINITY_DN1395_c0_g1 | FSG0038960 | 45.48 | 17.96 | 9.19 |
|  | SeC4H1 | TRINITY_DN2847_c0_g1 | FSG0025420 | 204.94 | 56.49 | 34.53 |
|  | Se4CL1 | TRINITY_DN12682_c0_g3 | FSG0047110 | 102.13 | 20.00 | 16.90 |
|  | Se4CL2 | TRINITY_DN8326_c0_g1 | FSG0140440 | 32.62 | 12.19 | 11.32 |
|  | Se4CL3 | TRINITY_DN9941_c2_g5 | FSG0262330 | 19.74 | 4.72 | 3.41 |
|  | SeCHS1 | TRINITY_DN6851_c0_g2 | FSG0130210 | 128.58 | 34.12 | 29.11 |
|  | SeDFR | TRINITY_DN7751_c0_g1 | FSG0149470 | 64.80 | 52.74 | 24.57 |
|  | SeF3H | TRINITY_DN1528_c0_g1 | FSG0060970 | 61.36 | 48.57 | 10.04 |
|  | SeFNSI1 | TRINITY_DN3320_c0_g1 | FSG0165280 | 6.47 | 2.31 | 0.01 |
|  | SeFLS1 | TRINITY_DN13123_c0_g1 | FSG0080030 | 350.68 | 180.13 | 18.18 |
|  | SeIFR1 | TRINITY_DN30670_c0_g1 | FSG0001040 | 174.20 | 67.55 | 49.21 |
|  | SeIFR2 | TRINITY_DN9432_c0_g2 | FSG0221220 | 11.38 | 3.89 | 0.06 |
|  | SeUGT1 | TRINITY_DN36036_c0_g1 | FSG0198300 | 23.96 | 7.35 | 3.13 |
|  | SeUGT2 | TRINITY_DN1817_c0_g1 | FSG0001140 | 457.00 | 117.31 | 22.31 |
|  | SeUGT3 | TRINITY_DN11664_c0_g1 | FSG0092180 | 88.73 | 7.99 | 1.27 |
|  | SeUGT4 | TRINITY_DN8312_c0_g1 | FSG0273210 | 220.28 | 197.24 | 129.05 |
| Negative regulation | Se4CL4 | TRINITY_DN3700_c0_g1 | FSG0062770 | 24.36 | 37.55 | 56.84 |
|  | Se4CL7 | TRINITY_DN1597_c0_g1 | FSG0054320 | 0.05 | 0.10 | 0.31 |
|  | Se4CL16 | TRINITY_DN29391_c0_g1 | FSG0111380 | 2.30 | 3.21 | 6.85 |
|  | SeFLS3 | TRINITY_DN5406_c0_g1 | FSG0197960 | 0.32 | 0.36 | 59.44 |
|  | SeUGT10 | TRINITY_DN4670_c0_g2 | FSG0092170 | 28.20 | 58.52 | 93.91 |
|  | SeUGT12 | TRINITY_DN20994_c0_g1 | FSG0054490 | 1.63 | 15.94 | 17.82 |
|  | SeUGT14 | TRINITY_DN9202_c0_g1 | FSG0049140 | 25.60 | 36.68 | 54.04 |
|  | SeUGT17 | TRINITY_DN5282_c0_g1 | FSG0097860 | 22.47 | 38.08 | 52.51 |
|  | SeUGT43 | TRINITY_DN1592_c0_g4 | FSG0077510 | 10.28 | 20.81 | 26.33 |
| Positive regulation | MYB | TRINITY_DN16426_c0_g1 | FSG0019590 | 52.97 | 32.15 | 0.96 |
|  |  | TRINITY_DN8224_c1_g1 | FSG0145900 | 47.28 | 30.52 | 4.35 |
|  |  | TRINITY_DN1268_c0_g1 | FSG0239060 | 83.35 | 78.7 | 14.39 |
|  |  | TRINITY_DN35951_c0_g1 | FSG0083540 | 105.43 | 8.21 | 0.2 |
|  |  | TRINITY_DN29547_c0_g1 | FSG0133380 | 85.64 | 72.27 | 41.93 |
|  |  | TRINITY_DN4220_c0_g1 | FSG0047650 | 40.83 | 5.75 | 0.6 |
|  |  | TRINITY_DN13548_c0_g1 | FSG0221650 | 20.35 | 4.57 | 0.1 |
|  |  | TRINITY_DN8224_c0_g1 | FSG0144630 | 44.32 | 31.73 | 24.13 |
|  |  | TRINITY_DN6596_c0_g2 | FSG0012660 | 12.31 | 9.19 | 1.84 |
|  |  | TRINITY_DN11004_c0_g1 | FSG0202580 | 15.45 | 11.32 | 4.82 |
|  |  | TRINITY_DN5504_c1_g1 | FSG0107850 | 19.34 | 10.51 | 9.28 |
|  |  | TRINITY_DN7801_c0_g1 | FSG0057100 | 33.53 | 29.45 | 21.6 |
| Negative regulation |  | TRINITY_DN11249_c0_g6 | FSG0036280 | 0.09 | 0.46 | 9.32 |
|  |  | TRINITY_DN22251_c0_g3 | FSG0078400 | 10.9 | 55.74 | 108.5 |
|  |  | TRINITY_DN2947_c0_g2 | FSG0027870 | 25.05 | 93.34 | 114.08 |
|  |  | TRINITY_DN1931_c1_g2 | FSG0031780 | 55.92 | 103.95 | 175.21 |
|  |  | TRINITY_DN3626_c0_g1 | FSG0079980 | 3.36 | 39.37 | 50.71 |
|  |  | TRINITY_DN28179_c0_g1 | FSG0250900 | 2.97 | 6.64 | 114.53 |
|  |  | TRINITY_DN1205_c0_g2 | FSG0135260 | 2.85 | 3.11 | 62.08 |
|  |  | TRINITY_DN1483_c0_g1 | FSG0186350 | 9.71 | 18.64 | 107.23 |
|  |  | TRINITY_DN528_c0_g6 | FSG0186350 | 0.43 | 1.3 | 25.99 |
|  |  | TRINITY_DN2310_c0_g1 | FSG0159340 | 1.48 | 20.54 | 156.59 |
|  |  | TRINITY_DN6637_c0_g3 | FSG0053890 | 19.45 | 25.09 | 63.59 |
|  |  | TRINITY_DN8447_c0_g1 | FSG0179850 | 2.65 | 13.35 | 18.17 |
| Positive regulation | bHLH | TRINITY_DN497_c0_g4 | FSG0231040 | 19.19 | 3.28 | 1.73 |
|  |  | TRINITY_DN8402_c0_g4 | FSG0034100 | 73.31 | 40.1 | 24.3 |
|  |  | TRINITY_DN1432_c0_g1 | FSG0242730 | 160.93 | 105.77 | 16.02 |
|  |  | TRINITY_DN2090_c0_g1 | FSG0267470 | 44.72 | 32.98 | 0.71 |
|  |  | TRINITY_DN34540_c0_g1 | FSG0002660 | 185.57 | 49.39 | 0.99 |
|  |  | TRINITY_DN10347_c0_g5 | FSG0125260 | 122.97 | 84.52 | 7.87 |
|  |  | TRINITY_DN2090_c0_g2 | FSG0207250 | 13.3 | 7.6 | 0.59 |
|  |  | TRINITY_DN93_c0_g1 | FSG0191090 | 30.05 | 15.94 | 7.19 |
| Negative regulation |  | TRINITY_DN15552_c0_g3 | FSG0136270 | 1.87 | 3.16 | 32.06 |
|  |  | TRINITY_DN3317_c3_g1 | FSG0093660 | 14.69 | 32 | 93.65 |
|  |  | TRINITY_DN7658_c1_g2 | FSG0166740 | 1.45 | 1.7 | 14.17 |
|  |  | TRINITY_DN3688_c0_g1 | FSG0074880 | 16.74 | 21.69 | 99.17 |
|  |  | TRINITY_DN497_c0_g3 | FSG0167820 | 16.78 | 17.45 | 36.61 |
|  |  | TRINITY_DN5333_c0_g1 | FSG0266210 | 10.99 | 24.98 | 35.19 |
| Positive regulation | WRKY | TRINITY_DN8546_c0_g2 | FSG0141310 | 19.38 | 9.63 | 5.04 |
| Negative regulation |  | TRINITY_DN10216_c0_g2 | FSG0039260 | 1.33 | 2.59 | 21.82 |
|  |  | TRINITY_DN6773_c1_g1 | FSG0220320 | 1.86 | 2.3 | 84.14 |
|  |  | TRINITY_DN3781_c0_g2 | FSG0124210 | 8.74 | 17.89 | 217.17 |
|  |  | TRINITY_DN693_c0_g2 | FSG0022640 | 34.61 | 66.13 | 366.37 |
|  |  | TRINITY_DN6927_c0_g1 | FSG0209270 | 2.87 | 7.86 | 98.98 |
|  |  | TRINITY_DN7191_c0_g1 | FSG0105690 | 0.89 | 2.56 | 27.59 |
|  |  | TRINITY_DN5594_c0_g1 | FSG0068420 | 13.18 | 24.57 | 78.61 |
|  |  | TRINITY_DN10216_c0_g1 | FSG0243340 | 1.39 | 6.32 | 35.39 |
|  |  | TRINITY_DN31454_c0_g1 | FSG0032540 | 0.51 | 2.41 | 13.7 |
| Positive regulation | bZIP | TRINITY_DN3268_c0_g2 | FSG0013290 | 34.42 | 5.87 | 1.15 |
|  |  | TRINITY_DN32450_c0_g1 | FSG0046700 | 16.93 | 15.38 | 3.75 |
|  |  | TRINITY_DN18271_c0_g1 | FSG0065450 | 10.37 | 8.93 | 3.92 |
|  |  | TRINITY_DN11492_c0_g3 | FSG0035590 | 3.63 | 1.47 | 0 |
|  |  | TRINITY_DN766_c1_g1 | FSG0254550 | 182.16 | 146.48 | 114.24 |
|  |  | TRINITY_DN3155_c0_g1 | FSG0213770 | 5.15 | 2.9 | 1.62 |
| Negative regulation |  | TRINITY_DN3931_c0_g1 | FSG0058440 | 18.57 | 71.18 | 112.92 |
|  |  | TRINITY_DN649_c2_g1 | FSG0058440 | 15.67 | 27.83 | 95.67 |
|  |  | TRINITY_DN253_c0_g2 | FSG0239400 | 9.3 | 9.72 | 28.61 |
|  |  | TRINITY_DN4526_c1_g1 | FSG0266940 | 19.42 | 36.82 | 65.21 |
|  |  | TRINITY_DN14257_c0_g2 | FSG0218820 | 2.57 | 3.05 | 8.26 |
| Positive regulation | Dof | TRINITY_DN7691_c1_g1 | FSG0107630 | 2.86 | 0.4 | 0.35 |
|  |  | TRINITY_DN10329_c2_g1 | FSG0264420 | 33.59 | 21.01 | 8.73 |
| Negative regulation |  | TRINITY_DN10329_c0_g2 | FSG0218970 | 0.1 | 1.02 | 149.5 |
|  |  | TRINITY_DN773_c0_g1 | FSG0179110 | 8.38 | 12.11 | 62.57 |
|  |  | TRINITY_DN10628_c0_g1 | FSG0107630 | 0.1 | 1.12 | 7.88 |
|  |  | TRINITY_DN10329_c0_g2 | FSG0218970 | 0.1 | 1.02 | 149.5 |
|  |  | TRINITY_DN773_c0_g1 | FSG0254550 | 8.38 | 12.11 | 62.57 |
|  |  | TRINITY_DN10628_c0_g1 | FSG0107630 | 0.1 | 1.12 | 7.88 |
