# Supplementary material for: Metabolic profiling and transcriptome analysis provide insights into the accumulation of flavonoids in chayote fruit during storage
Source: Front Nutr. 2023 Feb 27;10:1029745. doi: 10.3389/fnut.2023.1029745 (PMC10019507; doi:10.3389/fnut.2023.1029745)
Supplement: Supplementary file 11 [file Table_10.docx]

**Supplementary Table 10 |** The expression patterms in chayote fruits at three different storage stages (S1, S2, and S3) using qRT-PCR

| ID | S1-1 | S1-2 | S1-3 | S2-1 | S2-2 | S2-3 | S3-1 | S3-2 | S3-3 | S1 | S2 | S3 |
| --- | --- | --- | --- | --- | --- | --- | --- | --- | --- | --- | --- | --- |
| SePAL 2 | 1.87 | 2.16 | 2.07 | 1.73 | 1.26 | 1.67 | 0.33 | 0.79 | 0.41 | 2.03 | 1.55 | 0.51 |
| SeC4H 1 | 1.23 | 1.65 | 1.73 | 0.62 | 0.46 | 0.43 | 0.47 | 0.39 | 0.37 | 1.54 | 0.50 | 0.41 |
| Se4CL 2 | 2.40 | 2.47 | 2.03 | 1.93 | 1.77 | 1.65 | 1.59 | 1.56 | 1.48 | 2.30 | 1.78 | 1.54 |
| SeCHS 1 | 5.96 | 4.88 | 6.32 | 4.94 | 5.75 | 4.22 | 1.37 | 1.65 | 1.61 | 5.72 | 4.97 | 1.54 |
| SeUGT 1 | 1.86 | 1.57 | 1.64 | 1.77 | 1.62 | 1.41 | 1.61 | 1.58 | 1.42 | 1.69 | 1.60 | 1.54 |
| SeF3H | 4.12 | 4.38 | 3.66 | 2.27 | 2.15 | 2.18 | 1.24 | 1.08 | 1.17 | 4.05 | 2.20 | 1.16 |
| SeFLS 1 | 3.57 | 3.75 | 3.28 | 2.81 | 2.65 | 2.64 | 1.86 | 1.64 | 1.47 | 3.53 | 2.70 | 1.66 |
| SeIFR 2 | 1.44 | 1.18 | 1.21 | 1.17 | 1.08 | 1.12 | 0.89 | 1.22 | 0.95 | 1.28 | 1.12 | 1.02 |
| SeUGT 4 | 4.26 | 4.68 | 3.88 | 3.02 | 2.82 | 2.81 | 1.29 | 1.28 | 1.12 | 4.27 | 2.88 | 1.23 |
| SeDFR | 3.76 | 3.56 | 3.28 | 2.68 | 2.22 | 2.44 | 1.37 | 0.96 | 1.05 | 3.53 | 2.45 | 1.13 |
| SeIFR 1 | 1.24 | 1.33 | 0.82 | 0.37 | 0.46 | 0.52 | 0.41 | 0.33 | 0.26 | 1.13 | 0.45 | 0.33 |
| SeFNSI1 | 5.72 | 5.78 | 4.63 | 4.68 | 3.99 | 3.71 | 0.68 | 0.54 | 0.64 | 5.38 | 4.13 | 0.62 |
| SeFNSI2 | 4.57 | 4.46 | 4.52 | 4.27 | 3.31 | 3.85 | 6.73 | 7.78 | 7.19 | 4.52 | 3.81 | 7.23 |
| SeMYB1 | 2.51 | 2.88 | 3.17 | 2.12 | 2.08 | 2.06 | 2.04 | 1.88 | 0.96 | 2.85 | 2.09 | 1.63 |
| SeMYB2 | 3.44 | 4.21 | 3.09 | 3.01 | 2.22 | 2.74 | 1.34 | 1.09 | 1.51 | 3.58 | 2.66 | 1.31 |
| SeMYB3 | 9.31 | 9.25 | 8.86 | 2.13 | 2.68 | 3.02 | 2.11 | 1.97 | 1.82 | 9.14 | 2.61 | 1.97 |
| SebHLH1 | 1.92 | 2.01 | 1.81 | 1.65 | 1.74 | 1.79 | 0.40 | 0.68 | 0.83 | 1.91 | 1.73 | 0.64 |
| SebHLH2 | 4.10 | 4.62 | 3.87 | 1.96 | 1.63 | 1.72 | 0.31 | 0.44 | 0.56 | 4.20 | 1.77 | 0.44 |
