# Supplementary material for: Metabolic profiling and transcriptome analysis provide insights into the accumulation of flavonoids in chayote fruit during storage
Source: Front Nutr. 2023 Feb 27;10:1029745. doi: 10.3389/fnut.2023.1029745 (PMC10019507; doi:10.3389/fnut.2023.1029745)
Supplement: Supplementary file 13 [file Table_12.docx]

**Supplementary Table 12 |** Connection network between MYB and bHLH TFs and flavonoid metabolites

| TFs | Var1 | Var2 | cor | p_value |
| --- | --- | --- | --- | --- |
| MYB | metab_7173 | FSG0019590 | 0.883333333 | 0.0015905 |
|  | metab_10464 | FSG0019590 | 0.8 | 0.009627925 |
|  | metab_2581 | FSG0019590 | 0.866666667 | 0.002495398 |
|  | metab_5745 | FSG0019590 | 0.883333333 | 0.0015905 |
|  | metab_7269 | FSG0019590 | 0.933333333 | 0.0002359 |
|  | metab_8428 | FSG0019590 | 0.816666667 | 0.007224785 |
|  | metab_15535 | FSG0019590 | 0.866666667 | 0.002495398 |
|  | metab_7173 | FSG0145900 | 0.866666667 | 0.002495398 |
|  | metab_10464 | FSG0145900 | 0.833333333 | 0.005265691 |
|  | metab_7120 | FSG0145900 | 0.85 | 0.003704777 |
|  | metab_5745 | FSG0145900 | 0.9 | 0.000943062 |
|  | metab_7269 | FSG0145900 | 0.95 | 8.76E-05 |
|  | metab_15535 | FSG0145900 | 0.833333333 | 0.005265691 |
|  | metab_8428 | FSG0239060 | 0.916666667 | 0.000506619 |
|  | metab_7173 | FSG0083540 | 0.9 | 0.000943062 |
|  | metab_10464 | FSG0083540 | 0.85 | 0.003704777 |
|  | metab_7120 | FSG0083540 | 0.816666667 | 0.007224785 |
|  | metab_2581 | FSG0083540 | 0.8 | 0.009627925 |
|  | metab_5745 | FSG0083540 | 0.933333333 | 0.0002359 |
|  | metab_7269 | FSG0083540 | 0.933333333 | 0.0002359 |
|  | metab_15535 | FSG0083540 | 0.9 | 0.000943062 |
|  | metab_7173 | FSG0133380 | 0.916666667 | 0.000506619 |
|  | metab_10464 | FSG0133380 | 0.866666667 | 0.002495398 |
|  | metab_7120 | FSG0133380 | 0.833333333 | 0.005265691 |
|  | metab_5745 | FSG0133380 | 0.933333333 | 0.0002359 |
|  | metab_7269 | FSG0133380 | 0.983333333 | 1.94E-06 |
|  | metab_7173 | FSG0047650 | 0.866666667 | 0.002495398 |
|  | metab_10464 | FSG0047650 | 0.916666667 | 0.000506619 |
|  | metab_2581 | FSG0047650 | 0.866666667 | 0.002495398 |
|  | metab_5745 | FSG0047650 | 0.883333333 | 0.0015905 |
|  | metab_7269 | FSG0047650 | 0.933333333 | 0.0002359 |
|  | metab_7173 | FSG0144630 | 0.883333333 | 0.0015905 |
|  | metab_10464 | FSG0144630 | 0.833333333 | 0.005265691 |
|  | metab_7120 | FSG0144630 | 0.816666667 | 0.007224785 |
|  | metab_2581 | FSG0144630 | 0.816666667 | 0.007224785 |
|  | metab_5745 | FSG0144630 | 0.9 | 0.000943062 |
|  | metab_7269 | FSG0144630 | 0.95 | 8.76E-05 |
|  | metab_8428 | FSG0144630 | 0.816666667 | 0.007224785 |
|  | metab_15535 | FSG0144630 | 0.833333333 | 0.005265691 |
|  | metab_7173 | FSG0012660 | 0.9 | 0.000943062 |
|  | metab_10464 | FSG0012660 | 0.9 | 0.000943062 |
|  | metab_7120 | FSG0012660 | 0.816666667 | 0.007224785 |
|  | metab_2581 | FSG0012660 | 0.816666667 | 0.007224785 |
|  | metab_5745 | FSG0012660 | 0.866666667 | 0.002495398 |
|  | metab_7269 | FSG0012660 | 0.916666667 | 0.000506619 |
|  | metab_15535 | FSG0012660 | 0.866666667 | 0.002495398 |
|  | metab_7173 | FSG0202580 | 0.916666667 | 0.000506619 |
|  | metab_10464 | FSG0202580 | 0.883333333 | 0.0015905 |
|  | metab_2581 | FSG0202580 | 0.833333333 | 0.005265691 |
|  | metab_5745 | FSG0202580 | 0.883333333 | 0.0015905 |
|  | metab_7269 | FSG0202580 | 0.933333333 | 0.0002359 |
|  | metab_15535 | FSG0202580 | 0.85 | 0.003704777 |
|  | metab_10464 | FSG0107850 | 0.866666667 | 0.002495398 |
|  | metab_2581 | FSG0057100 | 0.866666667 | 0.002495398 |
|  | metab_10464 | FSG0057100 | 0.806865487 | 0.009846156 |
|  | metab_15535 | FSG0057100 | 0.816498456 | 0.006498465 |
|  | metab_7173 | FSG0057100 | 0.876546654 | 0.004678647 |
|  | metab_7269 | FSG0057100 | 0.804564612 | 0.006546132 |
|  | metab_7173 | FSG0036280 | -0.833333333 | 0.005265691 |
|  | metab_10464 | FSG0036280 | -0.966666667 | 2.16E-05 |
|  | metab_7120 | FSG0036280 | -0.916666667 | 0.000506619 |
|  | metab_5745 | FSG0036280 | -0.95 | 8.76E-05 |
|  | metab_7269 | FSG0036280 | -0.9 | 0.000943062 |
|  | metab_15535 | FSG0036280 | -0.866666667 | 0.002495398 |
|  | metab_10464 | FSG0078400 | -0.933333333 | 0.0002359 |
|  | metab_10464 | FSG0027870 | -0.916666667 | 0.000506619 |
|  | metab_7120 | FSG0031780 | -0.833333333 | 0.005265691 |
|  | metab_5745 | FSG0031780 | -0.85 | 0.003704777 |
|  | metab_7269 | FSG0031780 | -0.85 | 0.003704777 |
|  | metab_15535 | FSG0031780 | -0.966666667 | 2.16E-05 |
|  | metab_10464 | FSG0079980 | -0.8 | 0.009627925 |
|  | metab_10464 | FSG0250900 | -0.95 | 8.76E-05 |
|  | metab_5745 | FSG0250900 | -0.833333333 | 0.005265691 |
|  | metab_15535 | FSG0250900 | -0.8 | 0.009627925 |
|  | metab_7173 | FSG0186350 | -0.866666667 | 0.002495398 |
|  | metab_10464 | FSG0186350 | -0.95 | 8.76E-05 |
|  | metab_7120 | FSG0186350 | -0.9 | 0.000943062 |
|  | metab_5745 | FSG0186350 | -0.916666667 | 0.000506619 |
|  | metab_7269 | FSG0186350 | -0.916666667 | 0.000506619 |
|  | metab_15535 | FSG0186350 | -0.85 | 0.003704777 |
|  | metab_10464 | FSG0159340 | -0.866666667 | 0.002495398 |
|  | metab_2581 | FSG0159340 | -0.8 | 0.009627925 |
|  | metab_15535 | FSG0159340 | -0.8 | 0.009627925 |
|  | metab_7173 | FSG0053890 | -0.816666667 | 0.007224785 |
|  | metab_10464 | FSG0053890 | -0.883333333 | 0.0015905 |
|  | metab_7120 | FSG0053890 | -0.85 | 0.003704777 |
|  | metab_5745 | FSG0053890 | -0.866666667 | 0.002495398 |
|  | metab_7269 | FSG0053890 | -0.866666667 | 0.002495398 |
|  | metab_15535 | FSG0053890 | -0.933333333 | 0.0002359 |
|  | metab_10464 | FSG0179850 | -0.9 | 0.000943062 |
| bHLH | metab_10464 | FSG0231040 | 0.85 | 0.003704777 |
|  | metab_15484 | FSG0034100 | 0.866666667 | 0.002495398 |
|  | metab_7173 | FSG0034100 | 0.9 | 0.000943062 |
|  | metab_10464 | FSG0034100 | 0.883333333 | 0.0015905 |
|  | metab_648 | FSG0034100 | 0.8 | 0.009627925 |
|  | metab_7120 | FSG0034100 | 0.933333333 | 0.0002359 |
|  | metab_5745 | FSG0034100 | 0.916666667 | 0.000506619 |
|  | metab_7269 | FSG0034100 | 0.966666667 | 2.16E-05 |
|  | metab_8428 | FSG0034100 | 0.85 | 0.003704777 |
|  | metab_10826 | FSG0034100 | 0.866666667 | 0.002495398 |
|  | metab_15346 | FSG0034100 | 0.833333333 | 0.005265691 |
|  | metab_15535 | FSG0034100 | 0.8 | 0.009627925 |
|  | metab_15591 | FSG0034100 | 0.866666667 | 0.002495398 |
|  | metab_15484 | FSG0242730 | 0.816666667 | 0.007224785 |
|  | metab_7173 | FSG0242730 | 0.85 | 0.003704777 |
|  | metab_10464 | FSG0242730 | 0.816666667 | 0.007224785 |
|  | metab_648 | FSG0242730 | 0.883333333 | 0.0015905 |
|  | metab_7120 | FSG0242730 | 0.883333333 | 0.0015905 |
|  | metab_5745 | FSG0242730 | 0.866666667 | 0.002495398 |
|  | metab_7269 | FSG0242730 | 0.916666667 | 0.000506619 |
|  | metab_8428 | FSG0242730 | 0.85 | 0.003704777 |
|  | metab_10826 | FSG0242730 | 0.85 | 0.003704777 |
|  | metab_15535 | FSG0242730 | 0.883333333 | 0.0015905 |
|  | metab_15591 | FSG0242730 | 0.816666667 | 0.007224785 |
|  | metab_15484 | FSG0267470 | 0.866666667 | 0.002495398 |
|  | metab_7173 | FSG0267470 | 0.8 | 0.009627925 |
|  | metab_10464 | FSG0267470 | 0.816666667 | 0.007224785 |
|  | metab_7120 | FSG0267470 | 0.8 | 0.009627925 |
|  | metab_15484 | FSG0002660 | 0.866666667 | 0.002495398 |
|  | metab_10464 | FSG0002660 | 0.9 | 0.000943062 |
|  | metab_648 | FSG0002660 | 0.95 | 8.76E-05 |
|  | metab_7120 | FSG0002660 | 0.916666667 | 0.000506619 |
|  | metab_5745 | FSG0002660 | 0.9 | 0.000943062 |
|  | metab_7269 | FSG0002660 | 0.85 | 0.003704777 |
|  | metab_8428 | FSG0002660 | 0.933333333 | 0.0002359 |
|  | metab_10826 | FSG0002660 | 0.85 | 0.003704777 |
|  | metab_15346 | FSG0002660 | 0.816666667 | 0.007224785 |
|  | metab_15535 | FSG0002660 | 0.95 | 8.76E-05 |
|  | metab_15484 | FSG0125260 | 0.9 | 0.000943062 |
|  | metab_7173 | FSG0125260 | 0.816666667 | 0.007224785 |
|  | metab_10464 | FSG0125260 | 0.8 | 0.009627925 |
|  | metab_648 | FSG0125260 | 0.883333333 | 0.0015905 |
|  | metab_7120 | FSG0125260 | 0.85 | 0.003704777 |
|  | metab_5745 | FSG0125260 | 0.8 | 0.009627925 |
|  | metab_8428 | FSG0125260 | 0.8 | 0.009627925 |
|  | metab_15535 | FSG0125260 | 0.883333333 | 0.0015905 |
|  | metab_15484 | FSG0207250 | 0.816666667 | 0.007224785 |
|  | metab_7173 | FSG0207250 | 0.933333333 | 0.0002359 |
|  | metab_10464 | FSG0207250 | 0.9 | 0.000943062 |
|  | metab_648 | FSG0207250 | 0.8 | 0.009627925 |
|  | metab_7120 | FSG0207250 | 0.9 | 0.000943062 |
|  | metab_2581 | FSG0207250 | 0.8 | 0.009627925 |
|  | metab_5745 | FSG0207250 | 0.916666667 | 0.000506619 |
|  | metab_7269 | FSG0207250 | 0.966666667 | 2.16E-05 |
|  | metab_8428 | FSG0207250 | 0.866666667 | 0.002495398 |
|  | metab_10826 | FSG0207250 | 0.833333333 | 0.005265691 |
|  | metab_15346 | FSG0207250 | 0.833333333 | 0.005265691 |
|  | metab_15535 | FSG0207250 | 0.8 | 0.009627925 |
|  | metab_15591 | FSG0207250 | 0.883333333 | 0.0015905 |
|  | metab_15484 | FSG0191090 | 0.8 | 0.009627925 |
|  | metab_7173 | FSG0191090 | 0.866666667 | 0.002495398 |
|  | metab_10464 | FSG0191090 | 0.85 | 0.003704777 |
|  | metab_648 | FSG0191090 | 0.9 | 0.000943062 |
|  | metab_7120 | FSG0191090 | 0.866666667 | 0.002495398 |
|  | metab_2581 | FSG0191090 | 0.8 | 0.009627925 |
|  | metab_5745 | FSG0191090 | 0.85 | 0.003704777 |
|  | metab_7269 | FSG0191090 | 0.9 | 0.000943062 |
|  | metab_8428 | FSG0191090 | 0.883333333 | 0.0015905 |
|  | metab_10826 | FSG0191090 | 0.8 | 0.009627925 |
|  | metab_15535 | FSG0191090 | 0.9 | 0.000943062 |
|  | metab_15591 | FSG0191090 | 0.8 | 0.009627925 |
|  | metab_15484 | FSG0136270 | -0.916666667 | 0.000506619 |
|  | metab_10464 | FSG0136270 | -0.833333333 | 0.005265691 |
|  | metab_648 | FSG0136270 | -0.916666667 | 0.000506619 |
|  | metab_7120 | FSG0136270 | -0.916666667 | 0.000506619 |
|  | metab_5745 | FSG0136270 | -0.9 | 0.000943062 |
|  | metab_7269 | FSG0136270 | -0.816666667 | 0.007224785 |
|  | metab_8428 | FSG0136270 | -0.85 | 0.003704777 |
|  | metab_10826 | FSG0136270 | -0.883333333 | 0.0015905 |
|  | metab_15535 | FSG0136270 | -0.916666667 | 0.000506619 |
|  | metab_7173 | FSG0093660 | -0.816666667 | 0.007224785 |
|  | metab_648 | FSG0093660 | -0.9 | 0.000943062 |
|  | metab_7120 | FSG0093660 | -0.883333333 | 0.0015905 |
|  | metab_5745 | FSG0093660 | -0.866666667 | 0.002495398 |
|  | metab_7269 | FSG0093660 | -0.916666667 | 0.000506619 |
|  | metab_8428 | FSG0093660 | -0.85 | 0.003704777 |
|  | metab_10826 | FSG0093660 | -0.866666667 | 0.002495398 |
|  | metab_15535 | FSG0093660 | -0.9 | 0.000943062 |
|  | metab_15591 | FSG0093660 | -0.9 | 0.000943062 |
|  | metab_10464 | FSG0167820 | -0.8 | 0.009627925 |
|  | metab_10464 | FSG0266210 | -0.85 | 0.003704777 |
|  | metab_648 | FSG0266210 | -0.916666667 | 0.000506619 |
|  | metab_7120 | FSG0266210 | -0.833333333 | 0.005265691 |
|  | metab_5745 | FSG0266210 | -0.8 | 0.009627925 |
|  | metab_7269 | FSG0266210 | -0.8 | 0.009627925 |
|  | metab_8428 | FSG0266210 | -0.866666667 | 0.002495398 |
|  | metab_15535 | FSG0266210 | -0.916666667 | 0.000506619 |
|  | metab_10464 | FSG0124210 | -0.85 | 0.003704777 |
|  | metab_8428 | FSG0124210 | -0.866666667 | 0.002495398 |
|  | metab_7173 | FSG0209270 | -0.866666667 | 0.002495398 |
|  | metab_15484 | FSG0105690 | -0.816666667 | 0.007224785 |
|  | metab_7173 | FSG0105690 | -0.933333333 | 0.0002359 |
|  | metab_10464 | FSG0105690 | -0.9 | 0.000943062 |
|  | metab_648 | FSG0105690 | -0.8 | 0.009627925 |
|  | metab_7120 | FSG0105690 | -0.9 | 0.000943062 |
|  | metab_2581 | FSG0105690 | -0.8 | 0.009627925 |
|  | metab_5745 | FSG0105690 | -0.916666667 | 0.000506619 |
|  | metab_7269 | FSG0105690 | -0.966666667 | 2.16E-05 |
|  | metab_8428 | FSG0105690 | -0.866666667 | 0.002495398 |
|  | metab_10826 | FSG0105690 | -0.833333333 | 0.005265691 |
|  | metab_15346 | FSG0105690 | -0.833333333 | 0.005265691 |
|  | metab_15535 | FSG0105690 | -0.8 | 0.009627925 |
|  | metab_15591 | FSG0105690 | -0.883333333 | 0.0015905 |
|  | metab_15484 | FSG0068420 | -0.916666667 | 0.000506619 |
|  | metab_10464 | FSG0068420 | -0.916666667 | 0.000506619 |
|  | metab_7120 | FSG0068420 | -0.9 | 0.000943062 |
|  | metab_5745 | FSG0068420 | -0.883333333 | 0.0015905 |
|  | metab_8428 | FSG0068420 | -0.866666667 | 0.002495398 |
|  | metab_15346 | FSG0068420 | -0.8 | 0.009627925 |
|  | metab_10464 | FSG0243340 | -0.85 | 0.003704777 |
|  | metab_2581 | FSG0243340 | -0.8 | 0.009627925 |
|  | metab_7269 | FSG0243340 | -0.8 | 0.009627925 |
|  | metab_8428 | FSG0243340 | -0.833333333 | 0.005265691 |
