# Supplementary material for: Metabolic profiling and transcriptome analysis provide insights into the accumulation of flavonoids in chayote fruit during storage
Source: Front Nutr. 2023 Feb 27;10:1029745. doi: 10.3389/fnut.2023.1029745 (PMC10019507; doi:10.3389/fnut.2023.1029745)
Supplement: Supplementary file 14 [file Table_13.docx]

**Supplementary Table 13 |** The expression patterms in chayote fruits after treatment with phenylalanine at three different storage stages using qRT-PCR

| ID | CK1 | CK2 | CK3 | 0.5mmol/l | 0.5mmol/l | 0.5mmol/l | 1.0mmol/l | 1.0mmol/l | 1.0mmol/l |
| --- | --- | --- | --- | --- | --- | --- | --- | --- | --- |
| SePAL 2 | 1.17 | 1.05 | 0.78 | 1.03 | 1.23 | 1.25 | 2.06 | 2.05 | 2.36 |
| SeC4H 1 | 1.12 | 1.10 | 0.85 | 1.22 | 1.17 | 1.25 | 1.28 | 1.27 | 1.27 |
| Se4CL 2 | 0.84 | 0.96 | 1.21 | 2.62 | 2.54 | 2.60 | 5.25 | 4.88 | 4.95 |
| SeCHS 1 | 1.01 | 1.17 | 0.82 | 2.78 | 2.34 | 2.55 | 2.68 | 2.64 | 2.71 |
| SeUGT 1 | 0.92 | 1.21 | 0.88 | 2.15 | 2.31 | 2.24 | 3.64 | 3.35 | 3.48 |
| SeF3H | 1.22 | 0.85 | 1.04 | 3.17 | 3.18 | 3.06 | 4.45 | 4.17 | 4.05 |
| SeFLS 1 | 0.87 | 0.96 | 1.18 | 4.22 | 4.33 | 4.26 | 4.35 | 4.52 | 4.61 |
| SeIFR 2 | 1.02 | 1.07 | 0.90 | 1.20 | 1.31 | 1.24 | 4.00 | 3.56 | 4.32 |
| SeUGT 4 | 1.00 | 1.12 | 0.88 | 3.65 | 3.64 | 3.25 | 7.66 | 8.31 | 8.55 |
| SeDFR | 1.04 | 1.05 | 0.90 | 1.15 | 1.17 | 1.15 | 1.45 | 1.33 | 1.37 |
| SeIFR 1 | 1.11 | 1.15 | 0.74 | 1.66 | 1.71 | 1.85 | 2.01 | 1.94 | 2.23 |
| SeFNSI1 | 0.82 | 0.98 | 1.21 | 6.95 | 7.09 | 6.90 | 9.60 | 9.84 | 10.33 |
| SeFNSI2 | 1.31 | 0.78 | 0.91 | 2.18 | 2.05 | 1.98 | 5.56 | 5.37 | 5.84 |
| SeMYB1 | 1.01 | 1.03 | 0.96 | 5.36 | 5.44 | 5.25 | 10.21 | 9.99 | 10.17 |
| SeMYB2 | 0.87 | 0.95 | 1.18 | 1.32 | 1.89 | 1.52 | 9.12 | 7.27 | 8.89 |
| SeMYB3 | 1.02 | 0.82 | 1.16 | 5.91 | 4.81 | 4.23 | 5.33 | 4.97 | 6.03 |
| SebHLH1 | 1.19 | 1.10 | 0.71 | 1.29 | 1.74 | 1.79 | 4.79 | 3.24 | 3.21 |
| SebHLH2 | 0.96 | 0.88 | 1.16 | 3.74 | 3.33 | 3.54 | 6.44 | 7.87 | 8.24 |
